# Bridging innate immunity and iron-dependent death: the interplay between cyclic GMP–AMP synthase–stimulator of interferon genes nexus and ferroptosis in cancer and inflammation

**DOI:** 10.3389/fcell.2026.1766502

**Published:** 2026-03-06

**Authors:** Xin-xin Chen, Yun-qing Hou, Xin-xu Chen, Qi Zhou, Xiang Wang

**Affiliations:** 1 Department of Burn Surgery, The First Hospital of Jilin University, Jilin University, Changchun, China; 2 Department of Cardiovascular Disease, The First Hospital of Jilin University, Jilin University, Changchun, China; 3 Department of Anesthesiology, The First Hospital of Jilin University, Jilin University, Changchun, China; 4 State Key Laboratory of Organ Regeneration and Reconstruction, Institute of Zoology, Chinese Academy of Sciences, Beijing, China; 5 National Stem Cell Resource Center, Institute of Zoology, Chinese Academy of Sciences, Beijing, China; 6 Institute for Stem Cell and Regenerative Medicine, Chinese Academy of Sciences, Beijing, China; 7 Bejing Institute for Stem Cell and Regenerative Medicine, Beijing, China; 8 University of Chinese Academy of Sciences, Beijing, China

**Keywords:** cancer, cGAS–STING, ferroptosis, homeostasis, immunity

## Abstract

The cyclic GMP–AMP synthase (cGAS)–stimulator of interferon genes (STING) pathway and ferroptosis have emerged as fundamental biological mechanisms that converge in regulating cellular homeostasis and disease pathogenesis. As a central component of innate immunity, the cGAS–STING axis detects cytosolic double-stranded DNA through cGAS-mediated synthesis of 2′3′-cyclic GMP–AMP, subsequently triggering STING-dependent activation of the TBK1–IRF3 and nuclear factor-κB signaling cascades. Ferroptosis, an iron-catalyzed form of regulated cell death, is characterized by the accumulation of phospholipid hydroperoxide due to compromised antioxidant activity and dysregulated iron metabolism. Accumulating evidence has revealed the intricate crosstalk between these pathways. This review systematically explores the structural and biochemical bases of both pathways, identifies key bridging molecules that mediate their interactions, and discusses therapeutic strategies targeting this crosstalk, particularly in cancer treatment.

## 1 Introduction

The cyclic GMP–AMP synthase (cGAS)–stimulator of interferon (IFN) genes (STING) pathway and ferroptosis are two critical biological processes that have garnered significant attention in recent years for their roles in immunity, cell death, and pathogenesis. The cGAS–STING pathway is a cornerstone of innate immunity, primarily recognized for its ability to detect double-stranded DNA and initiate type I IFN responses against viral infections and cellular damage (Sun et al., 2013). In addition, the cGAS–STING pathway has emerged as a promising therapeutic target for various diseases, including cancer and autoimmune disorders (Hopfner and Hornung, 2020). By contrast, ferroptosis is a unique form of regulated cell death characterized by iron-dependent lipid peroxidation (Dixon et al., 2012). Ferroptosis was initially identified as a mechanism of cell death in cancer cells; however, it is now recognized as a fundamental, conserved cell death pathway implicated in various pathological processes, including ischemia/reperfusion injury, neurodegenerative diseases, and chronic inflammation (Yang and Stockwell, 2016).

Emerging evidence reveals a critical interaction between the cGAS–STING pathway and ferroptosis, a mechanism that couples innate immune sensing with iron-dependent cell death (Wang Y. et al., 2025). STING activation can promote ferroptosis by enhancing lipid peroxidation, disrupting glutathione (GSH) metabolism (Yu et al., 2022), and altering iron homeostasis (Zhao J. et al., 2024). Ferroptotic cell death releases double-stranded DNA, which activates cGAS–STING signaling, amplifying inflammatory and antitumor responses (Kim et al., 2023). A recent review comprehensively outlines the roles and mechanisms of cGAS–STING signaling pathway in ferroptosis, emphasizing their close disease-level interconnection (Ding et al., 2025); however, key nodal regulators remain uncharacterized.

In this review, we aim to provide a comprehensive overview of the cGAS–STING pathway and ferroptosis, focusing on their individual roles, shared regulatory networks, and potential therapeutic targets.

## 2 Overview of the cGAS–STING pathway and ferroptosis

### 2.1 Activation and feedback regulation of the cGAS–STING pathway

**FIGURE 1:**
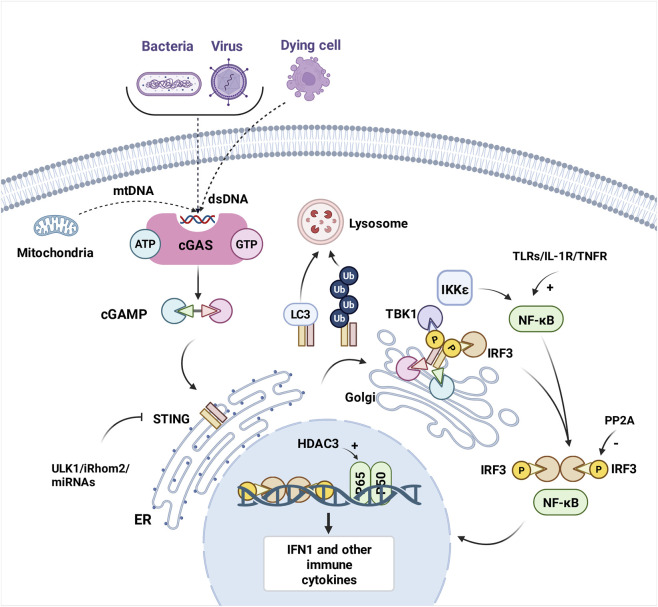
Illustration of key molecular events in the cGAS–STING signaling pathway. The cascade is initiated when cGAS senses exogenous or endogenous DNA, leading to STING activation. Subsequently, TBK1 is recruited and phosphorylated, which activates IRF3 through phosphorylation. Activated IRF3 and NF-κB translocate to the nucleus to induce expression of type I interferons and various proinflammatory cytokines. The diagram highlights negative feedback mechanisms, including autophagy- and ubiquitination-mediated STING degradation, which regulate pathway activity to maintain immune homeostasis. cGAMP, 2′3′-cyclic GMP–AMP; cGAS, cyclic GMP–AMP synthase; dsDNA, double-stranded DNA; ER, endoplasmic reticulum; HDAC3, histone deacetylase 3; IRF3, interferon regulatory factor 3; IFN, interferon; mtDNA, mitochondrial DNA; NF-κB, nuclear factor-κB; PP2A, protein phosphatase 2A; STING, stimulator of interferon genes; TBK1, TANK-binding kinase 1.

The cGAS–STING pathway serves as a crucial component of the innate immune system, functioning as a key bridge between DNA sensing and immune response (Chen and Xu, 2023). The activation and feedback regulation of the cGAS–STING pathway are summarized in Figure 1.

The pathway is initiated when cytosolic DNA, resulting from genomic damage or microbial infection (Sun et al., 2013), activates cGAS, an enzyme that catalyzes the synthesis of 2′3′-cyclic GMP–AMP (cGAMP) from ATP and GTP. cGAMP acts as an endogenous second messenger by binding to and activating STING, a sensor predominantly localized in the endoplasmic reticulum (Ishikawa and Barber, 2008; Wu et al., 2013). Upon cGAMP binding, the STING–cGAMP complex translocates from the endoplasmic reticulum to the Golgi apparatus, where STING oligomerization triggers the recruitment and activation of TANK-binding kinase 1 (TBK1) (Liu et al., 2021). TBK1 phosphorylates the C-terminal tail of STING, enabling its interaction with both TBK1 and IFN regulatory factor 3 (IRF3). Phosphorylated IRF3 forms dimers and translocates to the nucleus to induce type I IFN production (Zhang et al., 2019). In parallel, STING oligomers recruit inhibitors of the nuclear factor-κB (NF-κB) kinase subunit ε and activate NF-κB (Ritchie et al., 2022). Although NF-κB signaling can be TBK1-dependent, it does not strictly require TBK1 (Balka et al., 2020). The combined actions of IRF3 and NF-κB drive the expression of type I IFNs and other proinflammatory cytokines, which collectively orchestrate antiviral defense and inflammatory responses (Hopfner and Hornung, 2020; Ritchie et al., 2022).

Following the activation of the STING signaling pathway, a tightly regulated termination process is actively or passively engaged to prevent excessive or prolonged immune activation and maintain homeostasis. The pivotal mechanism in this process involves autophagy-mediated degradation: LC3-associated autophagy induced upon STING activation primarily functions as a host defense mechanism for the direct clearance of intracellular pathogens, during which STING is degraded (Gui et al., 2019); ubiquitination-mediated degradation: E3 ubiquitin ligases such as RNF5 and TRIM29 mediate the ubiquitination of STING, promoting its degradation (Zhong et al., 2009); dephosphorylation: phosphatases such as protein phosphatase 2A dephosphorylate TBK1 and IRF3, thereby terminating signal transduction (Liu et al., 2015); regulation by inhibitory proteins: cyclic dinucleotide produced by cGAS initiates the activation of UNC-51-like kinase, and STING is phosphorylated and IRF3 function suppressed (Konno et al., 2013); and post-transcriptional regulation: microRNAs or RNA-binding proteins can suppress STING expression by regulating the stability or translation efficiency of STING mRNA (Shah et al., 2019).

Beyond IFN signaling, STING activation also leads to its function as a proton channel (Liu et al., 2023; Xun et al., 2024). STING-induced cell death and ferroptosis are context-dependent but mechanistically related. As a proton channel, STING primarily triggers PANoptosis—involving pyroptosis, apoptosis, and necroptosis—during acute stress, yet it also promotes lipid peroxidation, a hallmark of ferroptosis (Liu et al., 2023). In contrast, chronic STING activation directly drives ferroptosis by downregulating CDKN2A/p16 and increasing oxidizable lipids through Acyl-coenzyme A Synthetase Long-Chain Family Member 4 (ACSL4)-dependent lipid peroxidation (Xun et al., 2024). Therefore, while STING-induced death is not synonymous with ferroptosis, STING can either directly trigger ferroptosis under chronic acvtivation or create a permissive environment for its execution during acute stress, highlighting STING as a critical node linking innate immune sensing to oxidative cell death fates.

### 2.2 Drivers of ferroptosis

**FIGURE 2:**
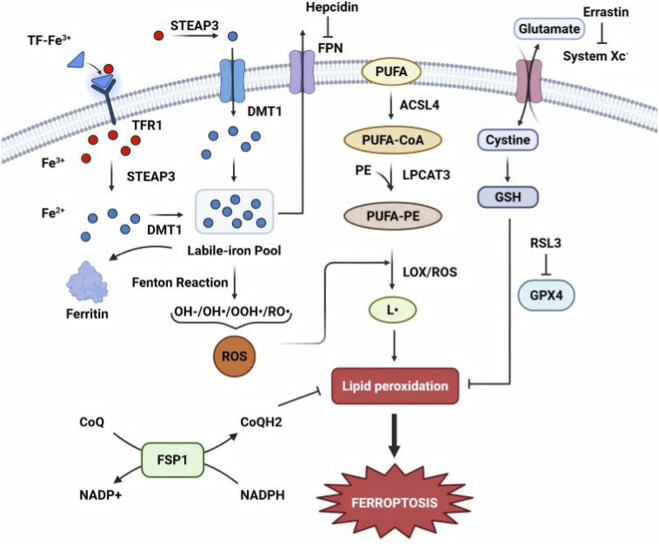
Mechanism of lipid peroxidation-mediated ferroptosis. Ferroptosis is initiated when labile iron (Fe^2+^) catalyzes Fenton reactions, generating reactive oxygen species (ROS) that oxidize polyunsaturated fatty acids (PUFAs) in membrane phospholipids, causing lethal lipid peroxidation. This process is exacerbated by dysregulated iron metabolism (e.g., hepcidin-mediated iron retention) and impaired antioxidant defenses, particularly inactivation of GPX4, which relies on glutathione to detoxify lipid peroxides, or the FSP1–CoQ10 system that scavenges lipid radicals. Accumulation of toxic lipid peroxidation products ultimately disrupts membrane integrity, leading to cell death. ACSL4, acyl-CoA synthetase long-chain family member 4; DMT1, divalent metal transporter 1; FPN, ferroportin; FSP1, ferroptosis suppressor protein 1; GPX4, glutathione peroxidase 4; GSH, glutathione; LPCAT3, lysophosphatidylcholine acyltransferase 3; PE, phosphatidylethanolamine; PUFA, polyunsaturated fatty acid; ROS, reactive oxygen species; STEAP3, six-transmembrane epithelial antigen of prostate 3; TF, transferrin; TFR1, transferrin receptor 1.

Ferroptosis is an iron-dependent, lipid peroxidation-driven form of programmed cell death. This primarily involves the key steps summarized in Figure 2. These processes ultimately result in cell membrane rupture and cell death (Dixon et al., 2012).

Iron plays a central role in ferroptosis, with its uptake, storage, and export being tightly regulated. Transferrin receptor 1 (TfR1) mediates iron uptake by internalizing transferrin-bound iron (Fe^3+^). Elevated TfR1 increases intracellular iron, thereby promoting ferroptosis. Ferritin, the primary iron storage protein, sequesters excess iron; its degradation (ferritinophagy) releases free iron, thereby exacerbating ferroptosis (Dixon et al., 2012). Iron regulatory proteins (IRP1/IRP2) control iron homeostasis by modulating TfR1 and ferritin expression. Ferroportin, the sole iron exporter, reduces intracellular iron. Its inhibition or degradation by hepcidin increases iron levels, thereby promoting ferroptosis (Ganz, 2013). Other molecules, such as Nrf2, counteract ferroptosis by upregulating ferritin and heme oxygenase 1 (Mancias et al., 2015), whereas p53 modulates iron metabolism genes (e.g., *SAT1*) to enhance lipid peroxidation (Jiang et al., 2015).

The Fenton reaction converts Fe^2+^ and H_2_O_2_ into hydroxyl radicals, which attack polyunsaturated fatty acids (PUFAs) in membranes, generating lipid radicals and peroxides (Su et al., 2019). Lipoxygenases directly oxidize PUFAs such as arachidonic acid and linoleic acid (Su et al., 2019). Additionally, oxidoreductases (NADPH-cytochrome P450 reductase and NADH-cytochrome b5 reductase) produce H_2_O_2_, further fueling the Fenton reaction (Yan et al., 2021). ACSL4 activates PUFAs by conjugating them to coenzyme A, whereas LPCAT3 incorporates them into phospholipids (e.g., phosphatidylethanolamine). These PUFA-rich lipids are highly susceptible to peroxidation, driving ferroptosis (Doll et al., 2017; Kagan et al., 2017).

The GSH–glutathione peroxidase 4 (GPX4) axis is the primary defense mechanism against ferroptosis. Erastin blocks cystine uptake *via* system xc^−^ (SLC7A11), depleting GSH and inactivating GPX4, thereby resulting in lethal lipid peroxide accumulation (Yang et al., 2014). GPX4 converts lipid hydroperoxides into harmless alcohols. Direct GPX4 inhibitors (e.g., RSL3) induce ferroptosis by allowing peroxide accumulation (Ursini and Maiorino, 2020). Ferroptosis suppressor protein one independently inhibits ferroptosis by reducing CoQ10 and vitamin K, which act as radical-trapping antioxidants (Bersuker et al., 2019; Mishima et al., 2022; Lv et al., 2023).

## 3 Crosstalk between the cGAS–STING pathway and ferroptosis: molecular mechanisms and functional consequences

STING, a molecule renowned for immune responses, has recently been found to exhibit an unexpected correlation with ferroptosis. Unraveling the molecules or pathways in this relationship may reveal novel therapeutic opportunities through immunogenic cell death mechanisms.

### 3.1 Link through mitochondria

#### 3.1.1 Mitochondria and ferroptosis

Morphological and functional changes in mitochondria confirm their central role in ferroptosis (Dixon et al., 2012). Elevated mitochondrial ROS promote ferroptosis by enhancing lipid peroxidation (Gan, 2021). Mitochondria-targeted antioxidants (e.g., MitoQ) attenuate this process (Jelinek et al., 2018; Fang et al., 2019).

Various mitochondrial proteins are involved in ferroptosis; therefore, multiple therapeutic strategies targeting mitochondria to enhance or alleviate ferroptosis have been developed. Mitochondrial PUFA-rich membranes are prime lipid peroxidation targets, with mitochondrial ACSL4 serving as a key metabolic regulator (Tao L. et al., 2024). Mitochondrial ferritin deficiency exacerbates ferroptosis in brain cells by elevating labile iron during ischemia/reperfusion injury (Wang P. et al., 2021). Knockdown of *SIRT3*, a deacetylase localized in the mitochondrial matrix, exacerbates iron overload and reactive oxygen species (ROS) production, resulting in ferroptosis (Feng et al., 2020). SFXN1 is a mitochondrial inner membrane protein. Under lipopolysaccharide (LPS) induction, NCOA4 mediates ferritinophagy, and the released Fe^2+^ activates SFXN1, leading to mitochondrial ROS accumulation and ferroptosis (Li et al., 2020). Voltage-dependent anion channels (VDACs) are abundant mitochondrial outer membrane proteins that facilitate the transport of small molecules. The VDAC1 oligomerization inhibitor VBIT 12 protects mitochondria, elevates ceramide and cardiolipin levels, and attenuates acetaminophen overdose-induced ferroptosis in hepatocytes (Niu et al., 2022). Inhibition of ceramide kinase modulates VDAC-associated mitochondrial function, inducing ferroptosis in KRAS-mutant non-small cell lung-cancer cells (Vu et al., 2022). In Parkinson’s disease, *Ndfip1* expression attenuates mitochondrial dysfunction by reducing VDAC levels and inhibiting ACSL4, thereby preventing neuronal ferroptosis (Fu et al., 2024). Dibutyl phthalate induces ferroptosis by upregulating and oligomerizing VDAC2, leading to mitochondria-dependent ferroptosis (Hou et al., 2024). FUNDC2, a mitochondrial outer membrane protein, interacts with SLC25A11 and plays a critical role in doxorubicin-induced ferroptosis in cardiomyocytes (Ta et al., 2022). Zinc-fluorouracil metallodrug networks induce mitochondrial ROS production, triggering ferroptosis in cancer cells (Lei et al., 2022). Polystyrene nanoplastics and cadmium inhibit the SIRT3–SOD2/GPX4 pathway, inducing oxidative stress and ferroptosis in ovarian mitochondria (Wu H. et al., 2025). ErZhiTianGui decoction maintains mitochondrial ROS levels and PINK1/Parkin-mediated mitochondrial homeostasis, preventing ferroptosis in aged mouse ovaries (Zhicheng et al., 2024). Astaxanthin stabilizes mitochondria membrane potential, mitigating interleukin (IL)-1β-induced ferroptosis in chondrocytes (Wang et al., 2022c).

#### 3.1.2 Mitochondria and STING

Regarding the role of mitochondria in the cGAS–STING pathway, X-box binding protein one modulates mitophagy, leading to mitochondrial DNA (mtDNA) leakage from macrophages, which activates cGAS–STING (Wang Q. et al., 2022). In a chronic arthritis model, tumor necrosis factor inhibited PINK1-mediated mitophagy, resulting in mtDNA accumulation and subsequent activation of cGAS–STING (Willemsen et al., 2021). In pressure-overloaded hearts, inducible nitric oxide synthase (iNOS) controls the release of mtDNA into the cytosol, which activates the cGAS–STING pathway and triggers sterile inflammation, ultimately leading to cardiac dysfunction. (Guo Y. et al., 2023). Beclin1-regulated autophagy protein one on cardiomyocyte-derived extracellular vehicles is reportedly necessary for extracellular vehicle-mediated mtDNA transfer to fibroblasts, subsequent activation of the cGAS–STING pathway, and induction of cardiac remodeling after ischemia/reperfusion injury (Zhang C. et al., 2023).

**FIGURE 3:**
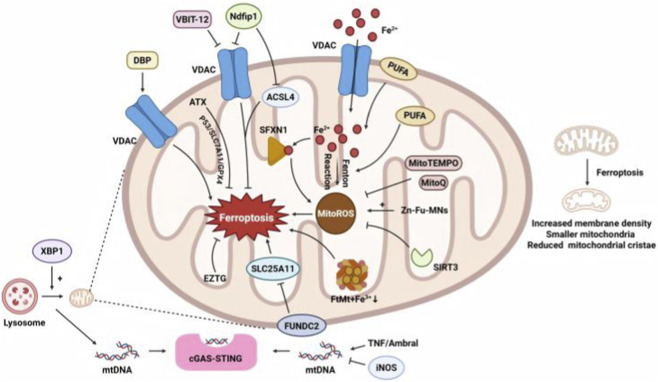
Role of mitochondria in ferroptosis and the cGAS–STING pathway. Mitochondrial dysfunction primes cells for ferroptosis by generating excessive ROS and disrupting iron homeostasis, leading to iron overload. Fe^2+^ catalyzes Fenton reactions that promote lipid peroxidation, particularly in mitochondrial membranes. Concurrently, mtDNA leakage activates the STING pathway, amplifying oxidative stress and inflammatory responses. The convergence of mitochondrial iron accumulation, uncontrolled lipid peroxidation, and STING-mediated inflammation drives ferroptotic cell death, as toxic lipid peroxides compromise membrane integrity and induce irreversible damage. ACSL4, acyl-CoA synthetase long-chain family member 4; DBP, dibutyl phthalate; EZTG, ErZhiTianGui; FUNDC2, FUN14 domain-containing 2; FtMt, mitochondrial ferritin; iNOS, inducible NO synthase; MitoQ, mitochondrial-targeted coenzyme Q10; mtDNA, mitochondrial DNA; Ndfip1, Nedd4 family interacting protein 1; PUFA, polyunsaturated fatty acid; SFXN1, siderofexin; SIRT3, sirtuin 3; TNF, tumor necrosis factor; VDACs, voltage-dependent anion channels; XBP1, X-box binding protein 1; Zn-Fu MNs, zinc-fluorouracil metallodrug networks.

Therefore, mitochondria serve as a crucial hub linking STING activation to ferroptosis through multiple mechanisms, including mtDNA leakage, mitochondrial ROS, and mitochondrial proteins (Figure 3). These interconnected pathways position mitochondria as both sensors of cellular stress (*via* STING) and executioners of ferroptosis, offering therapeutic targets for modulating inflammatory and cell death responses.

### 3.2 Link through p53

#### 3.2.1 p53 and STING

STING and p53 synergistically suppress tumors through their interconnected roles in innate immunity and tumor suppression. Wild-type p53 collaborates with the ubiquitin ligase TRIM24 to degrade DNA exonuclease TREX1, resulting in the accumulation of cytoplasmic DNA and activation of the cGAS/STING pathway (Ghosh et al., 2023). Additionally, upon activation of the DNA-binding protein IFI16, p53 —in conjunction with the DNA damage response factors ATM and poly (ADP-ribose) polymerase (PARP) 1—participates in STING signaling complex assembly, facilitating non-canonical STING activation (Dunphy et al., 2018). LRRC8C mediates cyclic dinucleotide transport *via* T-cell anion channels, activating STING–p53 signaling to suppress T-cell immunity (Concepcion et al., 2022).

However, mutations in p53 can reverse its immune regulatory effects. p53 mutations confer immunotherapy resistance in high-mutation tumors, reversible by STING agonists or p53 restoration (Zhu et al., 2023a). Mechanistically, mutant p53 binds to TBK1, preventing IRF3 activation and disrupting downstream signaling, thereby promoting tumor cell survival (Ghosh et al., 2021). In addition, p53 mutations impair cGAS–STING signaling but may lead to chromosomal instability and DNA fragment release, resulting in chronic low-level IFN-I inflammation (Qin et al., 2023). This further illuminates the complexity of upstream regulation within the STING pathway.

#### 3.2.2 p53 and ferroptosis

Recent research has revealed that p53-mediated regulation of ferroptosis is highly context-dependent, exhibiting bidirectional effects.

##### 3.2.2.1 p53 as a promoter of ferroptosis

The p53–SAT1 axis drives ALOX15-dependent ferroptosis *via* polyamine catabolism, inhibiting tumor growth (Ou et al., 2016). Another lipoxygenase family member, ALOX12, reportedly mediates ferroptosis *via* p53 in an ACSL4-independent manner (Chu et al., 2019). In the context of ferroptosis induced by erastin or RSL3, p53 targets ferredoxin reductase, leading to ferroptosis in a p53-dependent manner (Zhang et al., 2017). iPLA2β acts as a key repressor of p53-mediated ferroptosis, functioning independently of GPX4 in tumors (Chen et al., 2021).

##### 3.2.2.2 p53 as an inhibitor of ferroptosis

In addition, p53 acts to restrain ferroptosis; specifically, it limits erastin-induced ferroptosis *via* inhibiting dipeptidyl peptidase-4-dependent lipid peroxidation (Xie et al., 2017). The p53–p21 axis inhibits ferroptosis by preventing GSH depletion and reducing ROS accumulation (Tarangelo et al., 2018). Overexpression of Parkin, a p53 target gene, significantly eliminates mitochondria through mitophagy, thereby inhibiting ferroptosis (Zhang et al., 2011). p53 increases the expression of glutaminase 2, which plays a pivotal role in antioxidant defense; however, its precise role in ferroptosis requires further investigation (Hu et al., 2010).

**FIGURE 4:**
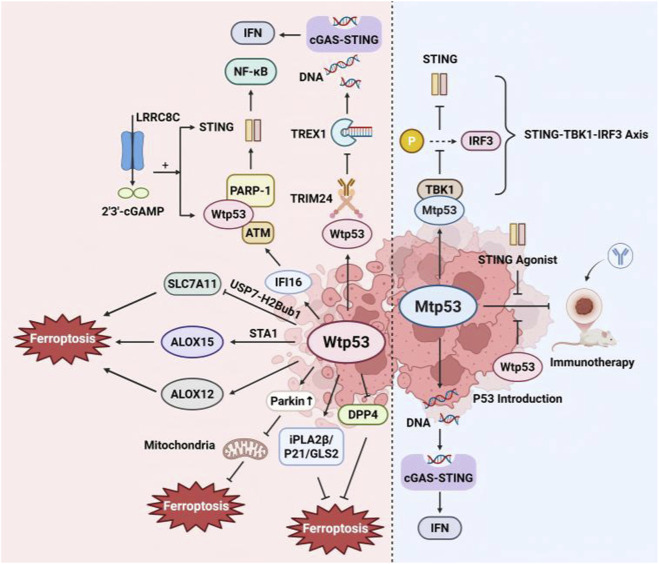
Role of p53 in ferroptosis and the cGAS–STING pathway. STING and p53 exert synergistic antitumor effects by coordinating immune activation and cell death pathways. p53 directly binds promoter regions of cGAS–STING genes, dynamically modulating their expression to enhance STING signaling. p53 regulates cytosolic DNA accumulation, the primary trigger for STING activation. Additionally, p53 controls ferroptosis *via* downstream effectors, including lipid peroxidation enzymes (e.g., ALOX15), ROS generation systems, and oxidative stress defense proteins (e.g., SLC7A11). This dual regulation promotes a therapeutic synergy: p53-mediated STING activation boosts antitumor immunity, whereas p53-driven ferroptosis eliminates cancer cells through iron-dependent lipid peroxidation. ALOX12, arachidonate 12-lipoxygenase; ALOX15, arachidonate 15-lipoxygenase; ATM, ataxia telangiectasia mutated; DPP4, dipeptidyl peptidase 4; GLS2, glutaminase 2; IFI16, interferon-γ-inducible factor 16; IRF3, interferon regulatory factor 3; LRRC8C, leucine-rich repeat-containing protein 8; Mtp53, mutant p53; PARP 1, poly (ADP-ribose) polymerase 1; SAT1, spermidine/spermine N1-acetyltransferase 1; TBK1, TANK-binding kinase 1; SLC7A11, solute carrier family 7 member 11; Wtp53, wild-type p53.

Therefore, p53 does not directly regulate STING-induced or STING-inhibited ferroptosis pathways but acts as an upstream modulator that fine-tunes immune responses through STING and governs cell-fate decisions *via* ferroptosis (Figure 4).

### 3.3 Link through the JAK–STAT pathway

#### 3.3.1 STAT1/2 and STING

STING activation triggers type I IFN production, which binds IFNAR1/2 to activate Janus kinase (JAK)1/TYK2 and initiate JAK–STAT signaling. Specifically, STAT2 forms a complex with STAT1 and IFN regulatory factor 9, also known as IFN-stimulated gene factor 3. This complex translocates to the nucleus, binds to IFN-stimulated response elements, and promotes the transcription of numerous IFN-stimulated genes. Additionally, STAT1 homodimers can form the IFN-γ-activated factor, which activates gene expression *via* the GAS promoter element. This process is integral to STING-initiated immune signaling and plays a critical role in the host’s defense against various immune challenges (Katze et al., 2002). Furthermore, type I IFNs induce STING expression through the STAT1 promoter binding site, creating a positive feedback loop that amplifies the immune response (Ma et al., 2015). Moreover, IFNs trigger the release of mtDNA into the cytoplasm, and JAK1-mediated phosphorylation of cGAS further activates the STING pathway (Tong et al., 2024).

The STING–JAK–STAT axis is regulated by multiple factors. The African swine fever virus pB318L protein suppresses host immunity by blocking STING Golgi translocation and STAT1/2 phosphorylation, inhibiting IFN-I/ISG production to enhance viral virulence (Liu et al., 2024). Loss of C9ORF72 results in increased STAT1 phosphorylation levels, which act upstream to further induce STING activation (Pang and Hu, 2023). Regarding therapeutic applications, dual PARP and histone deacetylase inhibitors activate the cGAS–STING and JAK–STAT pathways, boosting type I IFN responses in triple-negative breast cancer (TNBC) (Zhu Q. et al., 2024). Combined treatment with the farnesyltransferase inhibitor lonafarnib and the JAK–STAT inhibitor baricitinib suppresses the cGAS–STING–STAT1 pathway, reducing cytotoxicity and improving symptoms of Hutchinson–Gilford progeria syndrome (Arnold et al., 2021).

#### 3.3.2 STAT1/2 and ferroptosis

STAT1/2 regulates ferroptosis by modulating its key regulators. In tumors, hypoxia-induced HIF-2α/STAT1 upregulates ceruloplasmin mRNA in tumor-associated macrophages, which is transferred *via* extracellular vesicles to tumor cells, reducing iron accumulation and lipid peroxidation to confer ferroptosis resistance (Schwantes et al., 2024). Epigallocatechin-3-gallate inhibits the procancer effects of leptin by reducing STAT1 binding to the SLC7A11 promoter, thus facilitating ferroptosis (Li F. et al., 2023). Solamargine reduces STAT1 expression, downregulating mitochondrial carrier one and promoting ferroptosis in hepatocellular carcinoma (HCC) (Zhang et al., 2025). In tumor treatment, ionizing radiation stimulates ACSL4 and ferroptosis in intestinal cells *via* the STAT1/IRF1 axis (Kong et al., 2023). In cancer immunotherapy, mefloquine enhances PD-1 immunotherapy efficacy by upregulating LPCAT3 *via* the IFN-γ/STAT1–IRF1 axis, thereby promoting ferroptosis (Tao Q. et al., 2024). Conversely, CircPIAS1 inhibits STAT1 phosphorylation, activating the SLC7A11/GPX4 pathway and preventing IFN-induced ferroptosis in melanoma cells, revealing the mechanisms of immunotherapy resistance (Zang et al., 2024). Moreover, STAT2 mediates ferroptosis. In colorectal cancer, multilayer analysis has identified STAT2 as a potential upstream transcription factor that regulates ferroptosis (Zhong et al., 2022). In HCC, TMEM147 enhances STAT2-mediated DHCR7 expression, increasing 27-hydroxycholesterol levels to upregulate GPX4 and suppress ferroptosis (Huang et al., 2023).

In addition to tumor pathogenesis, STAT1 affects various organs by modulating ferroptosis. In the heart, peroxiredoxin 6 inhibits adriamycin-induced ferroptosis and heart failure by suppressing JAK2 and STAT1 phosphorylation, thus increasing SLC7A11, GPX4, and ferritin heavy chain levels (Xiong et al., 2024). STAT1 stimulates ferritin gene transcription, whereas opioid growth factor receptor inhibits STAT1 binding to ferritin promoters, preventing ferroptosis and providing cardioprotection to doxorubicin-treated patients (Chen X. et al., 2024). Crizotinib promotes STAT1 Ser727 phosphorylation, inhibiting Nrf2 transcription and inducing ferroptosis, thereby exacerbating liver injury (Guo et al., 2024). In the kidney, STAT1 induces macrophage ferroptosis by regulating the iron chaperone protein Pcbp1, promoting fibrosis in senescent kidneys (Wu et al., 2024). Liraglutide reduces STAT1 phosphorylation while increasing STAT3 and STAT6 phosphorylation, promoting M2 macrophage polarization and protecting against ferroptosis-induced renal dysfunction (Sun et al., 2024). Bicaudal D2 promotes IFN-γ signaling by facilitating STAT1 nuclear translocation, inhibiting SLC3A2 and SLC7A11 expression, and enhancing ferroptosis. Conversely, VHL degrades Bicaudal D2, mitigating renal injury (Hao et al., 2023). In Sjögren’s syndrome—a chronic autoimmune disorder in which the immune system attacks the body’s moisture-producing glands—elevated IFN-γ levels activate the JAK/STAT1 pathway, downregulating system xc^−^ components (e.g., SLC3A2, glutathione, and GPX4), leading to ferroptosis in salivary gland epithelial cells (Cao et al., 2023). In eye diseases, IFN-γ activates the JAK1-2/STAT1 pathway, which inhibits Fe^2+^ efflux protein, GPX4, and GSH, ultimately causing ferroptosis in human retinal pigment epithelial cells in age-related macular degeneration (Wei et al., 2022).

**FIGURE 5:**
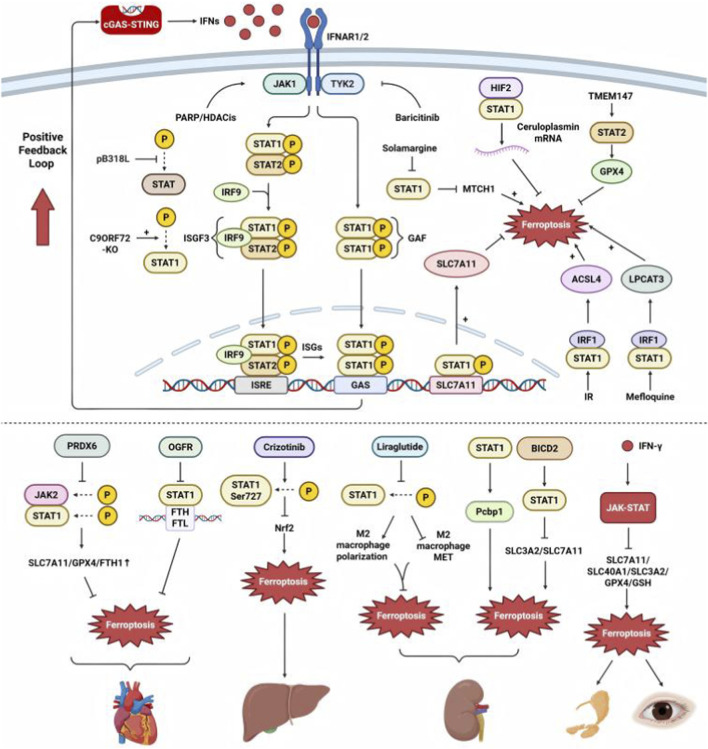
Mechanistic regulation of ferroptosis via the cGAS–STING–JAK–STAT1/2 pathway. The cGAS–STING pathway induces interferon (IFN) production, activating IFN receptors (IFNAR1/2) to initiate JAK–STAT signaling. Upon IFN binding, JAK1 and TYK2 phosphorylate STAT1 and STAT2, promoting transcription factor complex formation that binds interferon-stimulated response elements (ISREs) and gamma-activated sequence motifs. These complexes regulate key ferroptosis-related genes, thereby modulating lipid metabolism (ACSL4, LPCAT3), antioxidant defenses (SLC7A11, GPX4), and iron homeostasis/redox balance. This cascade culminates in ferroptotic cell death in multiple organs (heart, liver, kidney, eye), underscoring its physiological and pathological significance. BICD2, bicaudal D2; FTH, ferritin heavy chain; FTL, ferritin light chain; GSH, glutathione; HDAC, histone deacetylase; IFNAR1/2, IFNα/β receptor; IRF9, IFN regulatory factor 9; IR, ionizing radiation; ISGF3, IFN-stimulated gene factor 3; ISGs, interferon-stimulated genes; ISRE, interferon-stimulated response element; JAK, Janus kinase; LPCAT3, lysophosphatidylcholine acyltransferase 3; METs, macrophage extracellular traps; MTCH1, mitochondrial carrier 1; Nrf2, nuclear factor erythroid 2-related factor 2; OGFR, opioid growth factor receptor; PRDX6, peroxiredoxin 6; STAT, signal transducer and activator of transcription; TAK, tyrosine kinase; TMEM147, transmembrane protein 147.

Therefore, STAT1/2 serve as critical molecular bridges connecting STING-mediated immune responses with ferroptosis (Figure 5). Following STING activation, STAT1/2 regulate ferroptosis by modulating key regulators of ferroptosis, including SLC7A11, GPX4, ACSL4, ferritin, and LPCAT3. The STING-STAT1/2–ferroptosis nexus represents a therapeutic target, in which precise modulation could either enhance tumor immunogenicity or protect against tissue damage, depending on disease context.

#### 3.3.3 STAT3 and STING

STING and STAT3 exhibit reciprocal regulation, with STAT3 acting as a downstream effector of STING. In tumors with chromosomal instability, activation of cGAS–STING and non-canonical NF-κB pathway induces IL-6/STAT3 signaling, which paradoxically promotes tumor cell survival (Hong et al., 2022; Vasiyani et al., 2022), contrasting with the conventional understanding of STING as a tumor suppressor. Mechanistically, chemotherapy-induced DNA damage activates the STING–NF-κB pathway, leading to IL-6 production and STAT3 activation. This upregulates PD-L1 expression, mediating immunosuppression and enabling tumor cell survival (Vasiyani et al., 2022). All of these mechanisms coincide with the IL-6/JAK/STAT3 axis being a well-established pathway that suppresses antitumor immune responses (Johnson et al., 2018). STING-mediated STAT3 activation also plays a role in balancing cell survival and death, particularly in mitigating IFN/STAT1-associated cell death (Bakhoum, 2022). In addition to tumor biology, STING promotes macrophage chemokine production and migration *via* STAT3 activation, which reduces inflammation (Chen et al., 2025).

Regarding the feedback modulation of STAT3 on STING, STAT3 inhibition enhances STING signaling and antitumor immunity, suggesting a synergistic strategy in immunotherapy (Pei et al., 2019; Suter et al., 2021). In HCC, STAT3 deficiency potentiates the efficacy of sorafenib by activating the STING–IFN axis *via* apoptosis-induced DNA release (Wang et al., 2022b). Napabucasin, a STAT3 inhibitor, boosts cGAS–STING signaling *via* mitochondrial modulation to overcome tumor immune resistance (Wang et al., 2025). Additionally, AG490, a JAK2/STAT3 inhibitor, suppresses the cGAS/STING/NF-κB/p65 pathway, exacerbating neuronal senescence and inflammation post-stroke (Zhang W. et al., 2023). In metabolic disorders, STAT3 in pre-senescent adipocytes negatively regulates STAT1/cGAS–STING signaling, thus inhibiting cellular senescence and inflammation (Madani et al., 2021). IL-6 activates the JAK/STAT3 pathway, upregulating Jumonji domain-containing protein 3, which regulates *STING* gene expression and mediates inflammatory responses in diabetic wounds (Audu et al., 2022).

#### 3.3.4 STAT3 and ferroptosis

The regulatory relationship between STAT3 and ferroptosis is closely related to the expression of key ferroptosis-related proteins, including p53, ACSL4, GPX4, and SLC7A11. STAT3 inhibition promotes ferroptosis, suppresses tumor growth, and reduces chemotherapy resistance by upregulating p53 and directly downregulating GPX4 and SLC7A11 at the transcriptional level (Ouyang et al., 2022). STAT3 inhibits ACSL4 expression, thus suppressing ferroptosis (Wang Y. et al., 2021). Auranofin upregulates hepcidin *via* the NF-κB/IL-6/STAT3 pathway, thereby reducing ferroptosis (Yang et al., 2020). Chronic exposure of osteosarcoma cells to cisplatin hyperactivates the STAT3/Nrf2 signaling pathway, increasing GPX4 activity and inhibiting ferroptosis (Liu and Wang, 2019). The IL-6/JAK/STAT3 axis triggers tumor growth factor-β1 secretion from tumor-associated macrophages, activating GGT1 through the hepatic leukemia factor oncoprotein to suppress ferroptosis and drive TNBC progression (Li et al., 2022).

Considering the potential role of STAT3 in ferroptosis, several therapeutic strategies have been developed for cancer treatment. Thiostrepton, a STAT3 inhibitor, blocks GPX4 expression, inducing ferroptosis in pancreatic cancer cells (Zhang et al., 2022b). Ginsenoside Rh3 induces ferroptosis in rectal cancer cells by downregulating SLC7A11 *via* the STAT3/p53/NRF2 pathway (Wu et al., 2023). Bavastatin inhibits STAT3, upregulates p53, and downregulates SLC7A11, thereby promoting ferroptosis in osteosarcoma cells (Luo et al., 2021). In HCC, metformin downregulates ATF4 and inhibits STAT3 phosphorylation, promoting ferroptosis and enhancing sorafenib efficacy (Hu et al., 2023).

In contrast to its role in cancer, STAT3 exhibits a positive correlation with ferroptosis in noncancerous diseases. Inhibition of the IL-6/STAT3 pathway by Elabela activates xCT/GPX4, inhibiting ferroptosis in endothelial cells and offering therapeutic potential for hypertensive heart disease (Zhang Z. et al., 2022). STAT3 inhibition reduces NCOA4 and increases ferritin, protecting cardiomyocytes from high-fat diet-induced ferroptosis (Zhu et al., 2023b). In the liver, the inhibition of the AhR-STAT3-HO1/COX-2 pathway alleviates hepatocyte ferroptosis and improves human mesenchymal stem cell survival (Han et al., 2024). Lipocalin-2 activates the NF-κB/STAT3 pathway, promoting ferroptosis in hypoxic–ischemic brain damage (Luo et al., 2023). Additionally, STING drives ferroptosis by inhibiting STAT3 phosphorylation; *STING* knockdown mitigates LPS-induced ferroptosis *via* STAT3 suppression in lung epithelial cells (Gu et al., 2024).

**FIGURE 6:**
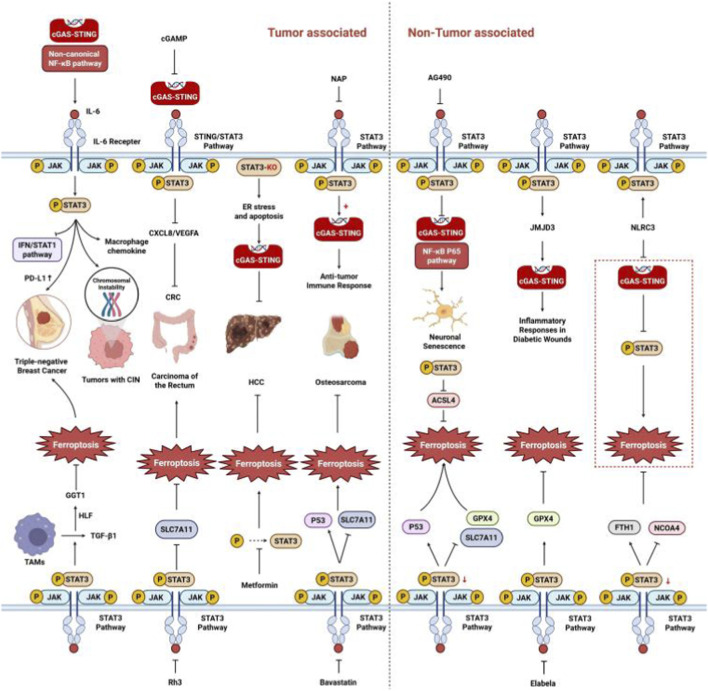
Regulatory interplay between cGAS–STING and ferroptosis *via* JAK/STAT3 signaling. The cGAS–STING pathway produces the second messenger cGAMP, which directly activates STAT3. STAT3, in turn, modulates cGAS–STING activity through multiple mechanisms, establishing a bidirectional regulatory loop. Within the nucleus, STAT3 regulates transcription of key ferroptosis-related genes, including tumor suppressor p53, GPX4, cystine/glutamate antiporter (SLC7A11 and ACSL4). Notably, the IL-6/JAK2/STAT3 axis, which is functionally linked to both cGAS–STING activation and ferroptosis regulation, is implicated in various pathological conditions such as neuropsychiatric disorders (depression), cerebrovascular disease (stroke), and metabolic disorders (diabetes). ACSL4, acyl-CoA synthetase long-chain family member 4; CRC, colorectal cancer; CIN, chromosomal instability; CXCL8, chemokine ligand 8; ER, endoplasmic reticulum; FTH1, ferritin heavy chain 1; GPX4, glutathione peroxidase 4; HCC, hepatocellular carcinoma; HLF, hepatic leukemia factor; JMJD3, Jumonji domain-containing protein 3; NAP, napabucasin; NCOA4, nuclear receptor coactivator 4; NLRC3, NLR family CARD domain-containing 3; Rh3, ginsenoside Rh3; TAMs, tumor-associated macrophages; TGF-β1, transforming growth factor beta 1; VEGFA, vascular endothelial growth factor A.

Therefore, STAT3 serves as a pivotal molecular switch that connects STING signaling to ferroptosis, exhibiting context-dependent dual functions (Figure 6).

### 3.4 Link through dihydroorotate dehydrogenase (DHODH)

#### 3.4.1 DHODH and ferroptosis

DHODH is a mitochondrial enzyme that is critical for maintaining mitochondrial membrane potential, regulating ROS production, and inhibiting ferroptosis in tumors. DHODH inactivation induces mitochondrial lipid peroxidation, particularly in cancer cells with low GPX4 expression. DHODH catalyzes the oxidation of dihydroorotic acid to orotate, while reducing coenzyme Q (CoQ) to CoQH2. The DHODH–CoQH2 system plays a key role in suppressing ferroptosis in cancer cells (Mao et al., 2021). In glioblastoma, deletion of proline-rich protein 11 increases lipid peroxidation and promotes ferroptosis in a DHODH-dependent manner (Miao et al., 2024). Inhibition of polymerase theta suppresses DHODH expression *via* the E2F4 transcription factor, increasing ferroptosis sensitivity and reducing stemness in gastric cancer cells (Peng et al., 2024).

Therapeutic strategies targeting DHODH in ferroptosis have been developed. Inhibition of DHODH synergizes with cisplatin and induces ferroptosis in cervical cancer cells (Jiang et al., 2023). Brequinar, a DHODH inhibitor, is modified with a mitochondrial targeting motif to enhance its anticancer activity by inducing ferroptosis (Hai et al., 2024). The nanomedicine ATO/SRF@BSA promotes mitochondrial lipid peroxidation by inhibiting GPX4 and downregulating the DHODH–CoQH2 system in TNBC (Zhou et al., 2024). SOD2 depletion and DHODH inhibition enhance the sensitivity of nasopharyngeal carcinoma cells to radiotherapy (Amos et al., 2023).

In a noncancer context, 17β-estradiol (E2) increases DHODH expression in the hippocampus, exerting neuroprotective effects against ferroptosis and addressing memory decline (Tian et al., 2023). In heart failure, E2 upregulates DHODH to inhibit ferroptosis, alleviating pathological hypertrophy and fibrosis (Wang et al., 2024). In aged donor hearts, reduced expression of cold-inducible RNA-binding protein leads to decreased DHODH levels, exacerbating ferroptosis in transplanted hearts (Zhu Y. et al., 2024). In diabetic cardiomyopathy, PACS2 upregulation leads to increased CPT1A expression, which inhibits DHODH and promotes cardiomyocyte ferroptosis (Xiang et al., 2024). In subarachnoid hemorrhage, menaquinone-4 activates SIRT1, which upregulates DHODH to attenuate ferroptosis (Zhang et al., 2024). DHODH inhibits the p53/ALOX15 pathway, ameliorating neuronal ferroptosis and spinal cord injury (Li D. et al., 2023). Under hypoxia, DHODH promotes cell survival through cooperation with GPX4 in corneal epithelial cell (Wu M. F. et al., 2025). In benzene-induced inflammatory anemia, DHODH interacts with IRP1 and ALOX12 to regulate ferroptosis (Zhang et al., 2022c). In septic mice, the absence of Lipocalin-2 increases DHODH expression, reducing ferroptosis and liver injury (Li et al., 2025).

#### 3.4.2 DHODH and STING

DHODH-induced ferroptosis promotes mitochondrial lipid peroxidation, leading to the release of mtDNA, which activates the cGAS–STING pathway, creating a feedback loop between ferroptosis and STING signaling. Notably, Mn^2+^ activates cGAS–STING to boost type I IFN, while inhibiting DHODH to trigger IFN-dependent ferroptosis *via* mitochondrial ROS and lipid peroxidation (Zhao et al., 2020; Zhang S. et al., 2022). In subarachnoid hemorrhage models, ginsenoside Rd inhibits the cGAS/STING pathway while activating DHODH, thereby suppressing neuronal ferroptosis (Jiang et al., 2025).

**FIGURE 7:**
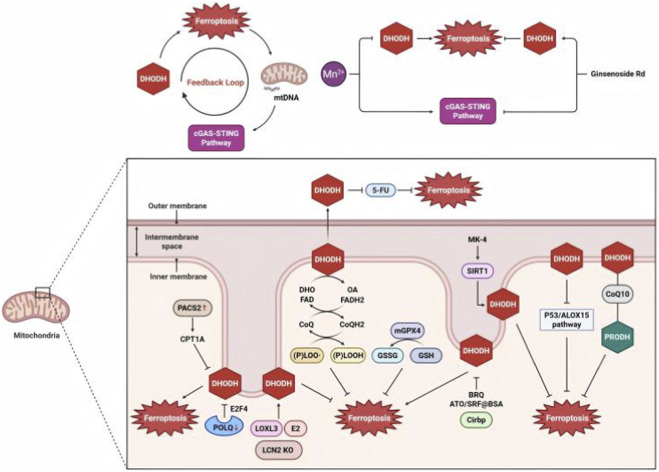
Mitochondrial DHODH-mediated ferroptosis regulation and its crosstalk with cGAS–STING signaling. In mitochondria, dihydroorotate dehydrogenase (DHODH) catalyzes the redox-dependent conversion of dihydroorotate (DHO) to orotate (OA) *via* FAD/FADH2 and CoQ/CoQH2 cycling. This metabolic process generates lipid peroxides [(P)LOO• and (P)LOOH], which induce ferroptosis. In cancer, DHODH interacts functionally with LCN2-mediated iron metabolism and the p53/ALOX15 lipid peroxidation pathway. In noncancer contexts, DHODH activity is modulated by metabolic regulators (CPT1A) and organelle trafficking factors (PACS2). Importantly, mtDNA released during ferroptosis links mitochondrial dysfunction to cGAS–STING activation. This network is further regulated by endogenous modulators like Mn^2+^ and SIRT1, as well as pharmacological agents such as ginsenoside Rd, brequinar (BRQ), arsenic trioxide/BSA nanocomplexes (ATO/SRF@BSA), and menaquinone-4 (MK-4). ATO, arsenic trioxide; BRQ, brequinar; Cirbp, cold-inducible RNA-binding protein; CoQ, coenzyme Q; CPT1A, carnitine palmitoyltransferase 1A; DHODH, dihydroorotate dehydrogenase; DHO, dihydroorotic acid; E2, 17β-estradiol; LOXL3, lysyl oxidase-like 3; mtDNA, mitochondrial DNA; KM-4, menaquinone-4; OA, orotate; PACS2, phosphofurin acidic cluster sorting protein 2; POLQ, polymerase theta; PRODH, proline dehydrogenase.

DHODH serves as a crucial mitochondrial regulator linking STING signaling to ferroptosis (Figure 7). By maintaining CoQH2 levels, DHODH suppresses lipid peroxidation and ferroptosis, while its inhibition triggers mitochondrial ROS accumulation and mtDNA release, activating cGAS–STING.

### 3.5 Other associations between STING and ferroptosis

STING reportedly promotes ferroptosis. Increased ICA69 activates the STING pathway, leading to cardiomyocyte ferroptosis (Kong et al., 2022). Moderate-intensity treadmill exercise reduces neuronal ferroptosis after traumatic brain injury by inhibiting STING (Chen J. et al., 2023).

STING promotes ferroptosis through its interaction with key regulators of mitochondrial dynamics and ferroptosis. STING promotes ferroptosis by binding to mitofusin 1/2, mediating mitofusion, which increases mitochondrial ROS and enhances ferroptosis (Li et al., 2021). In lupus nephritis, STING induces ferroptosis and inflammatory responses through TBK1/NF-κB-mediated downregulation of ACSL4, GPX4, and SLC7A11 (Chen J. et al., 2024). In diabetic nephropathy, STING inhibition stabilizes ferroportin 1, thereby attenuating ferroptosis (Zhao Q. X. et al., 2024). Hypertension induces mtDNA release from renal epithelial cells, activating STING, which interacts with ACSL4 to promote ferroptosis, renal inflammation, and fibrosis (Gao et al., 2023).

A complex bidirectional interaction has been demonstrated between key regulatory molecules of ferroptosis and the cGAS–STING pathway. GPX4 is essential for cGAS–STING signaling pathway activation, which is critical for the innate immune response against DNA viruses (Jia et al., 2020). In patients with chronic hepatitis B, reduced GPX4 expression in peripheral blood mononuclear cells during the immune tolerance period is associated with increased STING mRNA levels, suggesting a potential relationship between GPX4 and the cGAS–STING pathway (Su et al., 2024). GPX4 methylation modifications, activated by the cGAS–STING pathway, facilitate cancer immunotherapy (Chen B. et al., 2023). A high-iron diet or GPX4 depletion activates the STING pathway, leading to macrophage activation in pancreatic cancer (Dai et al., 2020). Ferroptosis increases cathepsin B expression, causing DNA damage and activating STING, creating a feedback loop in human pancreatic cancer cells (Kuang et al., 2020).

Therapies have been developed to evaluate STING activation and ferroptosis induction in various diseases. Manganese molybdate nanoparticles, containing MoO_4_^2–^ and Mn^2+^, activate the cGAS–STING pathway, promote IFN-γ secretion, and inhibit GPX4, thereby inducing ferroptosis in tumor cells (Lei et al., 2024). Mn(III)-SS-NEs activate the cGAS–STING pathway, secrete IFN-γ, and induce ferroptosis, creating an antitumor loop through manganese-induced immunogenic cell death (He et al., 2023). FeGd-HN@TA-Fe^2+^-SN38 nanoparticles activate STING and release Fe^2+^, inducing ferroptosis in tumor cells (Guo S. et al., 2023).

**FIGURE 8:**
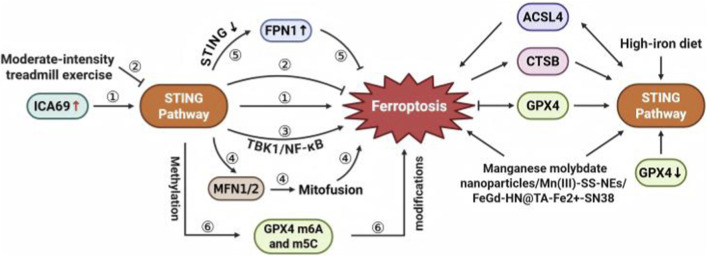
Additional pathways linking the cGAS–STING pathway and ferroptosis regulation. ICA69 protein upregulation activates STING signaling, promoting ferroptotic cell death (①). Moderate-intensity treadmill exercise suppresses STING activation, thereby inhibiting ferroptosis (②). STING-mediated activation of the TBK1/NF-κB cascade enhances ferroptosis execution by modulating key ferroptosis regulators (③). The STING pathway interacts with mitofusins (MFN1/2) to modulate mitochondrial fusion, resulting in ferroptosis (④). STING downregulation increases ferroportin 1 (FPN1) expression, inhibiting ferroptosis *via* iron efflux (⑤). STING activity influences glutathione peroxidase 4 (GPX4) modifications, altering its ferroptosis-suppressive function (⑥). Moreover, lipid metabolism enzymes (ACSL4), lysosomal proteases (CTSB), and antioxidant systems (GPX4) connect STING activity to ferroptosis regulation. CTSB, cathepsin B; FPN1, ferroportin 1; ICA69, islet cell autoantigen 69; MFN1/2, mitofusin 1/2; NF-κB, nuclear factor-κB; P62/SQSTM1, sequestosome 1; STING, stimulator of interferon genes; TBK1, TANK-binding kinase 1.

Therefore, the cGAS–STING pathway and ferroptosis engage in a complex bidirectional regulatory relationship with significant therapeutic implications (Figure 8).

## 4 Discussion

The intricate and context-dependent interplay between the cGAS–STING pathway and ferroptosis represents a sophisticated regulatory network that significantly affects cellular fate in pathological and physiological settings. Our synthesis of shared molecular mediators—such as mitochondrial dysfunction—highlights nodes of convergence that are not merely bystanders but active regulators of both pathways. These converging molecules present promising, yet complex, therapeutic targets for diseases where immune response and iron-driven cell death collide, such as in cancer, neurodegenerative disorders, and sterile inflammatory conditions. Consequently, our findings extend beyond the effects of individual pathways, revealing a novel intersection of immune and metabolic cell death.

While this review consolidates evidence linking key molecules to both pathways, the precision with which these shared elements coordinate cGAS–STING activation and ferroptosis remains incompletely mapped. For instance, it is still unclear whether STING activation preferentially sensitizes cells to ferroptosis in a spatiotemporally controlled manner. A key future direction involves the employment of live-cell imaging techniques to track the real-time transport of STING between organelles (such as the endoplasmic reticulum and mitochondria) and the generation of ferroptosis markers (such as GPX4 activity and lipid ROS), thereby elucidating the spatiotemporal coupling mechanisms between these processes. Furthermore, we observed the paradoxical phenomenon whereby p53 may both promote and inhibit ferroptosis. The present study hypothesizes an integrated model in which the intensity of intracellular stress signals and the cellular microenvironment jointly determine p53’s post-translational modification status and downstream pathways. Future research should systematically map p53 expression patterns under diverse stress conditions to decipher the key code governing this cell fate decision.

Looking forward, we propose that moving beyond observational linkage to mechanistic engineering of this interplay will open transformative therapeutic avenues. For example, in cancer therapy, designing combination regimens that sequentially activate STING and then trigger ferroptosis could maximize immunogenic cell death and antitumor immunity. Conversely, in inflammatory diseases, targeted disruption of specific branches of this network (e.g., by inhibiting lipid peroxidation at mitochondrial-ER contact sites) may mitigate pathological cGAS–STING activation without compromising overall immune function. The development of dual-acting agents or context-dependent modulators that can toggle between pro-death and pro-survival outcomes holds particular promise for precision medicine. Thus, unraveling the dynamic crosstalk between STING and ferroptosis will not only improve our understanding of cell death and immune signaling but also accelerate the translation of these insights into novel, pathway-aware therapeutics. It is noteworthy that, despite the promising mechanistic insights described here, key translational parameters for many of these agents remain poorly characterized. A substantial portion of the listed interventions is primarily supported by *in vitro* evidence, with limited *in vivo* data available on efficacy, toxicity, pharmacokinetics, or pharmacodynamics and minimal clinical validation to date. Therefore, further development of such therapeutics will require extensive additional preclinical and clinical research for robust validation.

In conclusion, this review presents the interplay between STING and ferroptosis as a dynamic and multifaceted relationship with profound implications for cancers and noncancerous diseases. By unraveling the molecular mechanisms and exploring innovative therapeutic approaches, researchers can harness the potential of this interplay to develop effective treatments and improve patient care.

## References

[B1] Amos A. Jiang N. Zong D. Gu J. Zhou J. Yin L. (2023). Depletion of SOD2 enhances nasopharyngeal carcinoma cell radiosensitivity *via* ferroptosis induction modulated by DHODH inhibition. BMC Cancer 23 (1), 117. 10.1186/s12885-022-10465-y 36737723 PMC9896811

[B2] Arnold R. Vehns E. Randl H. Djabali K. (2021). Baricitinib, a JAK-STAT inhibitor, reduces the cellular toxicity of the farnesyltransferase inhibitor lonafarnib in progeria cells. Int. J. Mol. Sci. 22 (14), 7474. 10.3390/ijms22147474 34299092 PMC8307450

[B3] Audu C. O. Melvin W. J. Joshi A. D. Wolf S. J. Moon J. Y. Davis F. M. (2022). Macrophage-specific inhibition of the histone demethylase JMJD3 decreases STING and pathologic inflammation in diabetic wound repair. Cell Mol. Immunol. 19 (11), 1251–1262. 10.1038/s41423-022-00919-5 36127466 PMC9622909

[B4] Bakhoum S. F. (2022). cGAS-STING and the deadly CIN: how chronic inflammation represents a therapeutic vulnerability in chromosomally unstable cancers. Trends Cancer 8 (10), 788–789. 10.1016/j.trecan.2022.07.006 35915014

[B5] Balka K. R. Louis C. Saunders T. L. Smith A. M. Calleja D. J. D’Silva D. B. (2020). TBK1 and IKKε act redundantly to mediate STING-induced NF-κB responses in myeloid cells. Cell Rep. 31 (1), 107492. 10.1016/j.celrep.2020.03.056 32268090

[B6] Bersuker K. Hendricks J. M. Li Z. Magtanong L. Ford B. Tang P. H. (2019). The CoQ oxidoreductase FSP1 acts parallel to GPX4 to inhibit ferroptosis. Nature 575 (7784), 688–692. 10.1038/s41586-019-1705-2 31634900 PMC6883167

[B7] Cao T. Zhou J. Liu Q. Mao T. Chen B. Wu Q. (2023). Interferon-γ induces salivary gland epithelial cell ferroptosis in Sjogren’s syndrome *via* JAK/STAT1-mediated inhibition of system Xc. Free Radic. Biol. Med. 205, 116–128. 10.1016/j.freeradbiomed.2023.05.027 37286044

[B8] Chen C. Xu P. (2023). Cellular functions of cGAS-STING signaling. Trends Cell Biol. 33 (8), 630–648. 10.1016/j.tcb.2022.11.001 36437149

[B9] Chen D. Chu B. Yang X. Liu Z. Jin Y. Kon N. (2021). iPLA2β-mediated lipid detoxification controls p53-driven ferroptosis independent of GPX4. Nat. Commun. 12 (1), 3644. 10.1038/s41467-021-23902-6 34131139 PMC8206155

[B10] Chen B. Hong Y. Zhai X. Deng Y. Hu H. Tian S. (2023a). m6A and m5C modification of GPX4 facilitates anticancer immunity *via* STING activation. Cell Death Dis. 14 (12), 809. 10.1038/s41419-023-06241-w 38065948 PMC10709592

[B11] Chen J. Zhu T. Yu D. Yan B. Zhang Y. Jin J. (2023b). Moderate intensity of treadmill exercise rescues TBI-induced ferroptosis, neurodegeneration, and cognitive impairments *via* suppressing STING pathway. Mol. Neurobiol. 60 (9), 4872–4896. 10.1007/s12035-023-03379-8 37193866 PMC10415513

[B12] Chen J. Chen P. Song Y. Wei J. Wu F. Sun J. (2024a). STING upregulation mediates ferroptosis and inflammatory response in lupus nephritis by upregulating TBK1 and activating NF-κB signal pathway. J. Biosci. 49, 9. 10.1007/s12038-023-00381-z 38186000

[B13] Chen X. Jian D. Xing J. Cheng X. Wang C. Wang C. (2024b). Targeting OGF/OGFR signal to mitigate doxorubicin-induced cardiotoxicity. Free Radic. Biol. Med. 223, 398–412. 10.1016/j.freeradbiomed.2024.08.005 39122201

[B14] Chen C. Cai X. Liu Z. Zhang W. Yang J. Tang Y. (2025). STING coordinates resolution of inflammation during wound repair by modulating macrophage trafficking through STAT3. J. Leukoc. Biol. 117 (3), qiae175. 10.1093/jleuko/qiae175 39119796

[B15] Chu B. Kon N. Chen D. Li T. Liu T. Jiang L. (2019). ALOX12 is required for p53-mediated tumour suppression through a distinct ferroptosis pathway. Nat. Cell Biol. 21 (5), 579–591. 10.1038/s41556-019-0305-6 30962574 PMC6624840

[B16] Concepcion A. R. Wagner L. E. Zhu J. Tao A. Y. Yang J. Khodadadi-Jamayran A. (2022). The volume-regulated anion channel LRRC8C suppresses T cell function by regulating cyclic dinucleotide transport and STING-p53 signaling. Nat. Immunol. 23 (2), 287–302. 10.1038/s41590-021-01105-x 35105987 PMC8991407

[B17] Dai E. Han L. Liu J. Xie Y. Zeh H. J. Kang R. (2020). Ferroptotic damage promotes pancreatic tumorigenesis through a TMEM173/STING-dependent DNA sensor pathway. Nat. Commun. 11 (1), 6339. 10.1038/s41467-020-20154-8 33311482 PMC7732843

[B18] Ding L. Zhang R. Du W. Wang Q. Pei D. (2025). The role of cGAS-STING signaling pathway in ferroptosis. J. Adv. Res. 76, 219–231. 10.1016/j.jare.2024.12.028 39710299 PMC12793764

[B19] Dixon S. J. Lemberg K. M. Lamprecht M. R. Skouta R. Zaitsev E. M. Gleason C. E. (2012). Ferroptosis: an iron-dependent form of nonapoptotic cell death. Cell 149 (5), 1060–1072. 10.1016/j.cell.2012.03.042 22632970 PMC3367386

[B20] Doll S. Proneth B. Tyurina Y. Y. Panzilius E. Kobayashi S. Ingold I. (2017). ACSL4 dictates ferroptosis sensitivity by shaping cellular lipid composition. Nat. Chem. Biol. 13 (1), 91–98. 10.1038/nchembio.2239 27842070 PMC5610546

[B21] Dunphy G. Flannery S. M. Almine J. F. Connolly D. J. Paulus C. Jønsson K. L. (2018). Non-canonical activation of the DNA sensing adaptor STING by ATM and IFI16 mediates NF-κB signaling after nuclear DNA damage. Mol. Cell 71 (5), 745–760.e5. 10.1016/j.molcel.2018.07.034 30193098 PMC6127031

[B22] Fang X. Wang H. Han D. Xie E. Yang X. Wei J. (2019). Ferroptosis as a target for protection against cardiomyopathy. Proc. Natl. Acad. Sci. U. S. A. 116 (7), 2672–2680. 10.1073/pnas.1821022116 30692261 PMC6377499

[B23] Feng X. Su H. He X. Chen J. X. Zeng H. (2020). SIRT3 deficiency sensitizes angiotensin-II-induced renal fibrosis. Cells 9 (11), 2510. 10.3390/cells9112510 33233553 PMC7699810

[B24] Fu X. Qu L. Xu H. Xie J. (2024). Ndfip1 protected dopaminergic neurons *via* regulating mitochondrial function and ferroptosis in Parkinson’s disease. Exp. Neurol. 375, 114724. 10.1016/j.expneurol.2024.114724 38365133

[B25] Gan B. (2021). Mitochondrial regulation of ferroptosis. J. Cell Biol. 220 (9), e202105043. 10.1083/jcb.202105043 34328510 PMC8329737

[B26] Ganz T. (2013). Systemic iron homeostasis. Physiol. Rev. 93 (4), 1721–1741. 10.1152/physrev.00008.2013 24137020

[B27] Gao L. Zhang J. Yang T. Jiang L. Liu X. Wang S. (2023). STING/ACSL4 axis-dependent ferroptosis and inflammation promote hypertension-associated chronic kidney disease. Mol. Ther. 31 (10), 3084–3103. 10.1016/j.ymthe.2023.07.026 37533255 PMC10556226

[B28] Ghosh M. Saha S. Bettke J. Nagar R. Parrales A. Iwakuma T. (2021). Mutant p53 suppresses innate immune signaling to promote tumorigenesis. Cancer Cell 39 (4), 494–508.e5. 10.1016/j.ccell.2021.01.003 33545063 PMC8044023

[B29] Ghosh M. Saha S. Li J. Montrose D. C. Martinez L. A. (2023). p53 engages the cGAS/STING cytosolic DNA sensing pathway for tumor suppression. Mol. Cell 83 (2), 266–280.e6. 10.1016/j.molcel.2022.12.023 36638783 PMC9993620

[B30] Gu Y. Lv L. Jin J. Hua X. Xu Q. Wu R. (2024). STING mediates LPS-induced acute lung injury by regulating ferroptosis. Exp. Cell Res. 438 (2), 114039. 10.1016/j.yexcr.2024.114039 38641125

[B31] Gui X. Yang H. Li T. Tan X. Shi P. Li M. (2019). Autophagy induction *via* STING trafficking is a primordial function of the cGAS pathway. Nature 567 (7747), 262–266. 10.1038/s41586-019-1006-9 30842662 PMC9417302

[B32] Guo S. Xiong W. Zhu J. Feng J. Zhou R. Fan Q. (2023a). A STING pathway-activatable contrast agent for MRI-guided tumor immunoferroptosis synergistic therapy. Biomaterials 302, 122300. 10.1016/j.biomaterials.2023.122300 37659110

[B33] Guo Y. You Y. Shang F. F. Wang X. Huang B. Zhao B. (2023b). iNOS aggravates pressure overload-induced cardiac dysfunction *via* activation of the cytosolic-mtDNA-mediated cGAS-STING pathway. Theranostics 13 (12), 4229–4246. 10.7150/thno.84049 37554263 PMC10405855

[B34] Guo L. Ma J. Xiao M. Liu J. Hu Z. Xia S. (2024). The involvement of the Stat1/Nrf2 pathway in exacerbating crizotinib-induced liver injury: implications for ferroptosis. Cell Death Dis. 15 (8), 600. 10.1038/s41419-024-06993-z 39160159 PMC11333746

[B35] Hai Y. Fan R. Zhao T. Lin R. Zhuang J. Deng A. (2024). A novel mitochondria-targeting DHODH inhibitor induces robust ferroptosis and alleviates immune suppression. Pharmacol. Res. 202, 107115. 10.1016/j.phrs.2024.107115 38423231

[B36] Han L. Ma C. Wu Z. Xu H. Li H. Pan G. (2024). AhR-STAT3-HO-1/COX-2 signalling pathway may restrict ferroptosis and improve hMSC accumulation and efficacy in mouse liver. Br. J. Pharmacol. 181 (1), 125–141. 10.1111/bph.16208 37538043

[B37] Hao W. Zhang H. Hong P. Zhang X. Zhao X. Ma L. (2023). Critical role of VHL/BICD2/STAT1 axis in crystal-associated kidney disease. Cell Death Dis. 14 (10), 680. 10.1038/s41419-023-06185-1 37833251 PMC10575931

[B38] He H. Du L. Xue H. An Y. Zeng K. Huang H. (2023). Triple tumor microenvironment-responsive ferroptosis pathways induced by manganese-based imageable nanoenzymes for enhanced breast cancer theranostics. Small Methods 7 (7), e2300230. 10.1002/smtd.202300230 37096886

[B39] Hong C. Schubert M. Tijhuis A. E. Requesens M. Roorda M. van den Brink A. (2022). cGAS-STING drives the IL-6-dependent survival of chromosomally instable cancers. Nature 607 (7918), 366–373. 10.1038/s41586-022-04847-2 35705809

[B40] Hopfner K. P. Hornung V. (2020). Molecular mechanisms and cellular functions of cGAS-STING signalling. Nat. Rev. Mol. Cell Biol. 21 (9), 501–521. 10.1038/s41580-020-0244-x 32424334

[B41] Hou T. Fan X. Zhang Q. Zhang H. Zhang D. Tao L. (2024). Dibutyl phthalate exposure induced mitochondria-dependent ferroptosis by enhancing VDAC2 in zebrafish ZF4 cells. Environ. Pollut. 348, 123846. 10.1016/j.envpol.2024.123846 38548160

[B42] Hu W. Zhang C. Wu R. Sun Y. Levine A. Feng Z. (2010). Glutaminase 2, a novel p53 target gene regulating energy metabolism and antioxidant function. Proc. Natl. Acad. Sci. U. S. A. 107 (16), 7455–7460. 10.1073/pnas.1001006107 20378837 PMC2867677

[B43] Hu Z. Zhao Y. Li L. Jiang J. Li W. Mang Y. (2023). Metformin promotes ferroptosis and sensitivity to sorafenib in hepatocellular carcinoma cells *via* ATF4/STAT3. Mol. Biol. Rep. 50 (8), 6399–6413. 10.1007/s11033-023-08492-4 37326750 PMC10374833

[B44] Huang J. Pan H. Sun J. Wu J. Xuan Q. Wang J. (2023). TMEM147 aggravates the progression of HCC by modulating cholesterol homeostasis, suppressing ferroptosis, and promoting the M2 polarization of tumor-associated macrophages. J. Exp. Clin. Cancer Res. 42 (1), 286. 10.1186/s13046-023-02865-0 37891677 PMC10612308

[B45] Ishikawa H. Barber G. N. (2008). STING is an endoplasmic reticulum adaptor that facilitates innate immune signalling. Nature 455 (7213), 674–678. 10.1038/nature07317 18724357 PMC2804933

[B46] Jelinek A. Heyder L. Daude M. Plessner M. Krippner S. Grosse R. (2018). Mitochondrial rescue prevents glutathione peroxidase-dependent ferroptosis. Free Radic. Biol. Med. 117, 45–57. 10.1016/j.freeradbiomed.2018.01.019 29378335

[B47] Jia M. Qin D. Zhao C. Chai L. Yu Z. Wang W. (2020). Redox homeostasis maintained by GPX4 facilitates STING activation. Nat. Immunol. 21 (7), 727–735. 10.1038/s41590-020-0699-0 32541831

[B48] Jiang L. Kon N. Li T. Wang S. J. Su T. Hibshoosh H. (2015). Ferroptosis as a p53-mediated activity during tumour suppression. Nature 520 (7545), 57–62. 10.1038/nature14344 25799988 PMC4455927

[B49] Jiang M. Song Y. Liu H. Jin Y. Li R. Zhu X. (2023). DHODH inhibition exerts synergistic therapeutic effect with cisplatin to induce ferroptosis in cervical cancer through regulating mTOR pathway. Cancers (Basel) 15 (2), 546. 10.3390/cancers15020546 36672495 PMC9856746

[B50] Jiang G. Y. Yang H. R. Li C. Liu N. Ma S. J. Jin B. X. (2025). Ginsenoside Rd alleviates early brain injury by inhibiting ferroptosis through cGAS/STING/DHODH pathway after subarachnoid hemorrhage. Free Radic. Biol. Med. 228, 299–318. 10.1016/j.freeradbiomed.2024.12.058 39746578

[B51] Johnson D. E. O’Keefe R. A. Grandis J. R. (2018). Targeting the IL-6/JAK/STAT3 signalling axis in cancer. Nat. Rev. Clin. Oncol. 15 (4), 234–248. 10.1038/nrclinonc.2018.8 29405201 PMC5858971

[B52] Kagan V. E. Mao G. Qu F. Angeli J. P. Doll S. Croix C. S. (2017). Oxidized arachidonic and adrenic PEs navigate cells to ferroptosis. Nat. Chem. Biol. 13 (1), 81–90. 10.1038/nchembio.2238 27842066 PMC5506843

[B53] Katze M. G. He Y. Gale M. Jr. (2002). Viruses and interferon: a fight for supremacy. Nat. Rev. Immunol. 2 (9), 675–687. 10.1038/nri888 12209136

[B54] Kim J. Kim H. S. Chung J. H. (2023). Molecular mechanisms of mitochondrial DNA release and activation of the cGAS-STING pathway. Exp. Mol. Med. 55 (3), 510–519. 10.1038/s12276-023-00965-7 36964253 PMC10037406

[B55] Kong C. Ni X. Wang Y. Zhang A. Zhang Y. Lin F. (2022). ICA69 aggravates ferroptosis causing septic cardiac dysfunction *via* STING trafficking. Cell Death Discov. 8 (1), 187. 10.1038/s41420-022-00957-y 35397620 PMC8994779

[B56] Kong P. Yang M. Wang Y. Yu K. N. Wu L. Han W. (2023). Ferroptosis triggered by STAT1- IRF1-ACSL4 pathway was involved in radiation-induced intestinal injury. Redox Biol. 66, 102857. 10.1016/j.redox.2023.102857 37611494 PMC10466894

[B57] Konno H. Konno K. Barber G. N. (2013). Cyclic dinucleotides trigger ULK1 (ATG1) phosphorylation of STING to prevent sustained innate immune signaling. Cell 155 (3), 688–698. 10.1016/j.cell.2013.09.049 24119841 PMC3881181

[B58] Kuang F. Liu J. Li C. Kang R. Tang D. (2020). Cathepsin B is a mediator of organelle-specific initiation of ferroptosis. Biochem. Biophys. Res. Commun. 533 (4), 1464–1469. 10.1016/j.bbrc.2020.10.035 33268027

[B59] Lei L. Dong Z. Xu L. Yang F. Yin B. Wang Y. (2022). Metal-fluorouracil networks with disruption of mitochondrion enhanced ferroptosis for synergistic immune activation. Theranostics 12 (14), 6207–6222. 10.7150/thno.75323 36168615 PMC9475458

[B60] Lei H. Li Q. Li G. Wang T. Lv X. Pei Z. (2024). Manganese molybdate nanodots with dual amplification of STING activation for “cycle” treatment of metalloimmunotherapy. Bioact. Mater. 31, 53–62. 10.1016/j.bioactmat.2023.07.026 37601278 PMC10432900

[B61] Li N. Wang W. Zhou H. Wu Q. Duan M. Liu C. (2020). Ferritinophagy-mediated ferroptosis is involved in sepsis-induced cardiac injury. Free Radic. Biol. Med. 160, 303–318. 10.1016/j.freeradbiomed.2020.08.009 32846217

[B62] Li C. Liu J. Hou W. Kang R. Tang D. (2021). STING1 promotes ferroptosis through MFN1/2-dependent mitochondrial fusion. Front. Cell Dev. Biol. 9, 698679. 10.3389/fcell.2021.698679 34195205 PMC8236825

[B63] Li H. Yang P. Wang J. Zhang J. Ma Q. Jiang Y. (2022). HLF regulates ferroptosis, development and chemoresistance of triple-negative breast cancer by activating tumor cell-macrophage crosstalk. J. Hematol. Oncol. 15 (1), 2. 10.1186/s13045-021-01223-x 34991659 PMC8740349

[B64] Li D. Lu X. Xu G. Liu S. Gong Z. Lu F. (2023a). Dihydroorotate dehydrogenase regulates ferroptosis in neurons after spinal cord injury *via* the P53-ALOX15 signaling pathway. CNS Neurosci. Ther. 29 (7), 1923–1939. 10.1111/cns.14150 36942513 PMC10324365

[B65] Li F. Hao S. Gao J. Jiang P. (2023b). EGCG alleviates obesity-exacerbated lung cancer progression by STAT1/SLC7A11 pathway and gut microbiota. J. Nutr. Biochem. 120, 109416. 10.1016/j.jnutbio.2023.109416 37451475

[B66] Li Y. Li L. Zhang Y. Yun Q. Du R. Ye H. (2025). Lipocalin-2 silencing alleviates sepsis-induced liver injury through inhibition of ferroptosis. Ann. Hepatol. 30 (1), 101756. 10.1016/j.aohep.2024.101756 39662594

[B67] Liu Q. Wang K. (2019). The induction of ferroptosis by impairing STAT3/Nrf2/GPx4 signaling enhances the sensitivity of osteosarcoma cells to cisplatin. Cell Biol. Int. 43 (11), 1245–1256. 10.1002/cbin.11121 30811078

[B68] Liu S. Cai X. Wu J. Cong Q. Chen X. Li T. (2015). Phosphorylation of innate immune adaptor proteins MAVS, STING, and TRIF induces IRF3 activation. Science 347 (6227), aaa2630. 10.1126/science.aaa2630 25636800

[B69] Liu N. Pang X. Zhang H. Ji P. (2021). The cGAS-STING pathway in bacterial infection and bacterial immunity. Front. Immunol. 12, 814709. 10.3389/fimmu.2021.814709 35095914 PMC8793285

[B70] Liu B. Carlson R. J. Pires I. S. Gentili M. Feng E. Hellier Q. (2023). Human STING is a proton channel. Science 381 (6657), 508–514. 10.1126/science.adf8974 37535724 PMC11260435

[B71] Liu X. Chen H. Ye G. Liu H. Feng C. Chen W. (2024). African swine fever virus pB318L, a trans-geranylgeranyl-diphosphate synthase, negatively regulates cGAS-STING and IFNAR-JAK-STAT signaling pathways. PLOS Pathog. 20 (4), e1012136. 10.1371/journal.ppat.1012136 38620034 PMC11018288

[B72] Luo Y. Gao X. Zou L. Lei M. Feng J. Hu Z. (2021). Bavachin induces ferroptosis through the STAT3/P53/SLC7A11 axis in osteosarcoma cells. Oxid. Med. Cell Longev. 2021, 1783485. 10.1155/2021/1783485 34707773 PMC8545544

[B73] Luo L. Deng L. Chen Y. Ding R. Li X. (2023). Identification of lipocalin 2 as a ferroptosis-related key gene associated with hypoxic-ischemic brain damage *via* STAT3/NF-κB signaling pathway. Antioxidants (Basel) 12 (1), 186. 10.3390/antiox12010186 36671050 PMC9854551

[B74] Lv Y. Liang C. Sun Q. Zhu J. Xu H. Li X. (2023). Structural insights into FSP1 catalysis and ferroptosis inhibition. Nat. Commun. 14 (1), 5933. 10.1038/s41467-023-41626-7 37739943 PMC10516921

[B75] Ma F. Li B. Yu Y. Iyer S. S. Sun M. Cheng G. (2015). Positive feedback regulation of type I interferon by the interferon-stimulated gene STING. EMBO Rep. 16 (2), 202–212. 10.15252/embr.201439366 25572843 PMC4328747

[B76] Madani A. Y. Majeed Y. Abdesselem H. B. Agha M. V. Vakayil M. Sukhun N. K. A. (2021). Signal transducer and activator of transcription 3 (STAT3) suppresses STAT1/interferon signaling pathway and inflammation in senescent preadipocytes. Antioxidants (Basel) 10 (2), 334. 10.3390/antiox10020334 33672392 PMC7927067

[B77] Mancias J. D. Pontano Vaites L. Nissim S. Biancur D. E. Kim A. J. Wang X. (2015). Ferritinophagy *via* NCOA4 is required for erythropoiesis and is regulated by iron dependent HERC2-mediated proteolysis. eLife 4, e10308. 10.7554/eLife.10308 26436293 PMC4592949

[B78] Mao C. Liu X. Zhang Y. Lei G. Yan Y. Lee H. (2021). DHODH-mediated ferroptosis defence is a targetable vulnerability in cancer. Nature 593 (7860), 586–590. 10.1038/s41586-021-03539-7 33981038 PMC8895686

[B79] Miao Z. Xu L. Gu W. Ren Y. Li R. Zhang S. (2024). A targetable PRR11-DHODH axis drives ferroptosis- and temozolomide-resistance in glioblastoma. Redox Biol. 73, 103220. 10.1016/j.redox.2024.103220 38838551 PMC11179629

[B80] Mishima E. Ito J. Wu Z. Nakamura T. Wahida A. Doll S. (2022). A non-canonical vitamin K cycle is a potent ferroptosis suppressor. Nature 608 (7924), 778–783. 10.1038/s41586-022-05022-3 35922516 PMC9402432

[B81] Niu B. Lei X. Xu Q. Ju Y. Xu D. Mao L. (2022). Protecting mitochondria *via* inhibiting VDAC1 oligomerization alleviates ferroptosis in acetaminophen-induced acute liver injury. Cell Biol. Toxicol. 38 (3), 505–530. 10.1007/s10565-021-09624-x 34401974

[B82] Ou Y. Wang S. J. Li D. Chu B. Gu W. (2016). Activation of SAT1 engages polyamine metabolism with p53-mediated ferroptotic responses. Proc. Natl. Acad. Sci. U. S. A. 113 (44), E6806–E6812. 10.1073/pnas.1607152113 27698118 PMC5098629

[B83] Ouyang S. Li H. Lou L. Huang Q. Zhang Z. Mo J. (2022). Inhibition of STAT3-ferroptosis negative regulatory axis suppresses tumor growth and alleviates chemoresistance in gastric cancer. Redox Biol. 52, 102317. 10.1016/j.redox.2022.102317 35483272 PMC9108091

[B84] Pang W. Hu F. (2023). C9ORF72 suppresses JAK-STAT mediated inflammation. iScience 26 (5), 106579. 10.1016/j.isci.2023.106579 37250330 PMC10214391

[B85] Pei J. Zhang Y. Luo Q. Zheng W. Li W. Zeng X. (2019). STAT3 inhibition enhances CDN-induced STING signaling and antitumor immunity. Cancer Lett. 450, 110–122. 10.1016/j.canlet.2019.02.029 30790684

[B86] Peng Y. Zheng W. Chen Y. Lei X. Yang Z. Yang Y. (2024). POLQ inhibition attenuates the stemness and ferroptosis resistance in gastric cancer cells *via* downregulation of dihydroorotate dehydrogenase. Cell Death Dis. 15 (4), 248. 10.1038/s41419-024-06618-5 38575587 PMC10995193

[B87] Qin Z. Liu H. Sheng Q. Dan J. Wu X. Li H. (2023). Mutant p53 leads to low-grade IFN-I-induced inflammation and impairs cGAS-STING signalling in mice. Eur. J. Immunol. 53 (9), e2250211. 10.1002/eji.202250211 37377275

[B88] Ritchie C. Carozza J. A. Li L. (2022). Biochemistry, cell biology, and pathophysiology of the innate immune cGAS-cGAMP-STING pathway. Annu. Rev. Biochem. 91, 599–628. 10.1146/annurev-biochem-040320-101629 35287475

[B89] Schwantes A. Wickert A. Becker S. Baer P. C. Weigert A. Brüne B. (2024). Tumor associated macrophages transfer ceruloplasmin mRNA to fibrosarcoma cells and protect them from ferroptosis. Redox Biol. 71, 103093. 10.1016/j.redox.2024.103093 38382185 PMC10900931

[B90] Shah A. U. Cao Y. Siddique N. Lin J. Yang Q. (2019). miR29a and miR378b influence CpG-stimulated dendritic cells and regulate cGAS/STING pathway. Vaccines (Basel) 7 (4), 197. 10.3390/vaccines7040197 31779082 PMC6963666

[B91] Su L. J. Zhang J. H. Gomez H. Murugan R. Hong X. Xu D. (2019). Reactive oxygen species-induced lipid peroxidation in apoptosis, autophagy, and ferroptosis. Oxid. Med. Cell Longev. 2019, 5080843. 10.1155/2019/5080843 31737171 PMC6815535

[B92] Su X. Wang Z. Li J. Gao S. Fan Y. Wang K. (2024). Hypermethylation of the glutathione peroxidase 4 gene promoter is associated with the occurrence of immune tolerance phase in chronic hepatitis B. Virol. J. 21 (1), 72. 10.1186/s12985-024-02346-6 38515187 PMC10958902

[B93] Sun L. Wu J. Du F. Chen X. Chen Z. J. (2013). Cyclic GMP-AMP synthase is a cytosolic DNA sensor that activates the type I interferon pathway. Science 339 (6121), 786–791. 10.1126/science.1232458 23258413 PMC3863629

[B94] Sun Z. Zhang F. Gao Z. Wu J. Bi Q. Zheng X. (2024). Liraglutide alleviates ferroptosis in renal ischemia reperfusion injury *via* inhibiting macrophage extracellular trap formation. Int. Immunopharmacol. 142 (B), 113258. 10.1016/j.intimp.2024.113258 39340991

[B95] Suter M. A. Tan N. Y. Thiam C. H. Khatoo M. MacAry P. A. Angeli V. (2021). cGAS-STING cytosolic DNA sensing pathway is suppressed by JAK2-STAT3 in tumor cells. Sci. Rep. 11 (1), 7243. 10.1038/s41598-021-86644-x 33790360 PMC8012641

[B96] Ta N. Qu C. Wu H. Zhang D. Sun T. Li Y. (2022). Mitochondrial outer membrane protein FUNDC2 promotes ferroptosis and contributes to doxorubicin-induced cardiomyopathy. Proc. Natl. Acad. Sci. U. S. A. 119 (36), e2117396119. 10.1073/pnas.2117396119 36037337 PMC9457330

[B97] Tao L. Xue Y. F. Sun F. F. He X. Wang H. Q. Tong C. C. (2024a). MitoQ protects against carbon tetrachloride-induced hepatocyte ferroptosis and acute liver injury by suppressing mtROS-mediated ACSL4 upregulation. Toxicol. Appl. Pharmacol. 486, 116914. 10.1016/j.taap.2024.116914 38522585

[B98] Tao Q. Liu N. Wu J. Chen J. Chen X. Peng C. (2024b). Mefloquine enhances the efficacy of anti-PD-1 immunotherapy *via* IFN-γ-STAT1-IRF1-LPCAT3-induced ferroptosis in tumors. J. Immunother. Cancer 12 (3), e008554. 10.1136/jitc-2023-008554 38471712 PMC10936479

[B99] Tarangelo A. Magtanong L. Bieging-Rolett K. T. Li Y. Ye J. Attardi L. D. (2018). p53 suppresses metabolic stress-induced ferroptosis in cancer cells. Cell Rep. 22 (3), 569–575. 10.1016/j.celrep.2017.12.077 29346757 PMC5791910

[B100] Tian Y. Xie Y. Guo Z. Feng P. You Y. Yu Q. (2023). 17β-oestradiol inhibits ferroptosis in the hippocampus by upregulating DHODH and further improves memory decline after ovariectomy. Redox Biol. 62, 102708. 10.1016/j.redox.2023.102708 37116254 PMC10163677

[B101] Tong Z. Zou J. P. Wang S. Y. Luo W. W. Wang Y. Y. (2024). Activation of the cGAS-STING-IRF3 axis by type I and II interferons contributes to host defense. Adv. Sci. (Weinh) 11 (35), e2308890. 10.1002/advs.202308890 39004913 PMC11425201

[B102] Ursini F. Maiorino M. (2020). Lipid peroxidation and ferroptosis: the role of GSH and GPx4. Free Radic. Biol. Med. 152, 175–185. 10.1016/j.freeradbiomed.2020.02.027 32165281

[B103] Vasiyani H. Mane M. Rana K. Shinde A. Roy M. Singh J. (2022). DNA damage induces STING mediated IL-6-STAT3 survival pathway in triple-negative breast cancer cells and decreased survival of breast cancer patients. Apoptosis 27 (11-12), 961–978. 10.1007/s10495-022-01763-8 36018392

[B104] Vu N. T. Kim M. Stephenson D. J. MacKnight H. P. Chalfant C. E. (2022). Ceramide kinase inhibition drives ferroptosis and sensitivity to cisplatin in mutant KRAS lung cancer by dysregulating VDAC-mediated mitochondria function. Mol. Cancer Res. 20 (9), 1429–1442. 10.1158/1541-7786.Mcr-22-0085 35560154 PMC9444881

[B105] Wang P. Cui Y. Ren Q. Yan B. Zhao Y. Yu P. (2021a). Mitochondrial ferritin attenuates cerebral ischaemia/reperfusion injury by inhibiting ferroptosis. Cell Death Dis. 12 (5), 447. 10.1038/s41419-021-03725-5 33953171 PMC8099895

[B106] Wang Y. Zhao Y. Ye T. Yang L. Shen Y. Li H. (2021b). Ferroptosis signaling and regulators in atherosclerosis. Front. Cell Dev. Biol. 9, 809457. 10.3389/fcell.2021.809457 34977044 PMC8716792

[B107] Wang Q. Bu Q. Liu M. Zhang R. Gu J. Li L. (2022a). XBP1-mediated activation of the STING signalling pathway in macrophages contributes to liver fibrosis progression. JHEP Rep. 4 (11), 100555. 10.1016/j.jhepr.2022.100555 36185574 PMC9520276

[B108] Wang X. Hu R. Song Z. Zhao H. Pan Z. Feng Y. (2022b). Sorafenib combined with STAT3 knockdown triggers ER stress-induced HCC apoptosis and cGAS-STING-mediated anti-tumor immunity. Cancer Lett. 547, 215880. 10.1016/j.canlet.2022.215880 35981569

[B109] Wang X. Liu Z. Peng P. Gong Z. Huang J. Peng H. (2022c). Astaxanthin attenuates osteoarthritis progression *via* inhibiting ferroptosis and regulating mitochondrial function in chondrocytes. Chem. Biol. Interact. 366, 110148. 10.1016/j.cbi.2022.110148 36084724

[B110] Wang C. Chen C. Zhou J. Shi J. Sun H. Li J. (2024). DHODH alleviates heart failure *via* the modulation of CoQ-related ferroptotic inhibition. Front. Biosci. (Landmark Ed.) 29 (7), 267. 10.31083/j.fbl2907267 39082362

[B111] Wang L. Bi S. Li Z. Liao A. Li Y. Yang L. (2025). Napabucasin deactivates STAT3 and promotes mitoxantrone-mediated cGAS-STING activation for hepatocellular carcinoma chemo-immunotherapy. Biomaterials 313, 122766. 10.1016/j.biomaterials.2024.122766 39180916

[B112] Wang Y. Wu S. Wang Y. Wang C. Zheng W. Yun X. (2025a). Interplay of cGAS-STING and ferroptosis: crosstalk, molecular mechanisms, and therapeutic prospects. Arch. Toxicol. 99 (12), 4883–4905. 10.1007/s00204-025-04150-9 40828197

[B113] Wei T. T. Zhang M. Y. Zheng X. H. Xie T. H. Wang W. Zou J. (2022). Interferon-γ induces retinal pigment epithelial cell Ferroptosis by a JAK1-2/STAT1/SLC7A11 signaling pathway in age-related macular degeneration. FEBS J. 289 (7), 1968–1983. 10.1111/febs.16272 34741776

[B114] Willemsen J. Neuhoff M. T. Hoyler T. Noir E. Tessier C. Sarret S. (2021). TNF leads to mtDNA release and cGAS/STING-dependent interferon responses that support inflammatory arthritis. Cell Rep. 37 (6), 109977. 10.1016/j.celrep.2021.109977 34758308

[B115] Wu J. Sun L. Chen X. Du F. Shi H. Chen C. (2013). Cyclic GMP-AMP is an endogenous second messenger in innate immune signaling by cytosolic DNA. Science 339 (6121), 826–830. 10.1126/science.1229963 23258412 PMC3855410

[B116] Wu Y. Pi D. Zhou S. Yi Z. Dong Y. Wang W. (2023). Ginsenoside Rh3 induces pyroptosis and ferroptosis through the Stat3/p53/NRF2 axis in colorectal cancer cells. Acta Biochim. Biophys. Sin. (Shanghai) 55 (4), 587–600. 10.3724/abbs.2023068 37092860 PMC10195137

[B117] Wu L. Lin H. Li S. Huang Y. Sun Y. Shu S. (2024). Macrophage iron dyshomeostasis promotes aging-related renal fibrosis. Aging Cell 23 (11), e14275. 10.1111/acel.14275 39016438 PMC11561705

[B118] Wu H. Feng L. Wu H. Wang L. Xu H. Fu F. (2025a). Synergistic effects of PS-NPs and Cd on ovarian toxicity in adolescent rats: ferroptosis by induction of mitochondrial redox imbalance *via* the SIRT3-SOD2/Gpx4 pathway. Ecotoxicol. Environ. Saf. 290, 117622. 10.1016/j.ecoenv.2024.117622 39732061

[B119] Wu M. F. Peng X. Zhang M. C. Guo H. Xie H. T. (2025b). Ferroptosis and PANoptosis under hypoxia pivoting on the crosstalk between DHODH and GPX4 in corneal epithelium. Free Radic. Biol. Med. 228, 173–182. 10.1016/j.freeradbiomed.2024.12.050 39761766

[B120] Xiang H. Lyu Q. Chen S. Ouyang J. Xiao D. Liu Q. (2024). PACS2/CPT1A/DHODH signaling promotes cardiomyocyte ferroptosis in diabetic cardiomyopathy. Cardiovasc. Diabetol. 23 (1), 432. 10.1186/s12933-024-02514-6 39633391 PMC11619700

[B121] Xie Y. Zhu S. Song X. Sun X. Fan Y. Liu J. (2017). The tumor suppressor p53 limits ferroptosis by blocking DPP4 activity. Cell Rep. 20 (7), 1692–1704. 10.1016/j.celrep.2017.07.055 28813679

[B122] Xiong J. Zhou R. Deng X. (2024). PRDX6 alleviated heart failure by inhibiting doxorubicin-induced ferroptosis through the JAK2/STAT1 pathway inactivation. Vitro Cell Dev. Biol. Anim. 60 (4), 354–364. 10.1007/s11626-024-00889-0 38530594

[B123] Xun J. Zhang Z. Lv B. Lu D. Yang H. Shang G. (2024). A conserved ion channel function of STING mediates noncanonical autophagy and cell death. EMBO Rep. 25 (2), 544–569. 10.1038/s44319-023-00045-x 38177926 PMC10897221

[B124] Yan B. Ai Y. Sun Q. Ma Y. Cao Y. Wang J. (2021). Membrane damage during ferroptosis is caused by oxidation of phospholipids catalyzed by the oxidoreductases POR and CYB5R1. Mol. Cell 81 (2), 355–369.e10. 10.1016/j.molcel.2020.11.024 33321093

[B125] Yang W. S. Stockwell B. R. (2016). Ferroptosis: death by lipid peroxidation. Trends Cell Biol. 26 (3), 165–176. 10.1016/j.tcb.2015.10.014 26653790 PMC4764384

[B126] Yang W. S. SriRamaratnam R. Welsch M. E. Shimada K. Skouta R. Viswanathan V. S. (2014). Regulation of ferroptotic cancer cell death by GPX4. Cell 156 (1-2), 317–331. 10.1016/j.cell.2013.12.010 24439385 PMC4076414

[B127] Yang L. Wang H. Yang X. Wu Q. An P. Jin X. (2020). Auranofin mitigates systemic iron overload and induces ferroptosis *via* distinct mechanisms. Signal Transduct. Target Ther. 5 (1), 138. 10.1038/s41392-020-00253-0 32732975 PMC7393508

[B128] Yu X. Zhu D. Luo B. Kou W. Cheng Y. Zhu Y. (2022). IFNγ enhances ferroptosis by increasing JAK-STAT pathway activation to suppress SLCA711 expression in adrenocortical carcinoma. Oncol. Rep. 47 (5), 97. 10.3892/or.2022.8308 35322867 PMC8968764

[B129] Zang X. He X. Y. Xiao C. M. Lin Q. Wang M. Y. Liu C. Y. (2024). Circular RNA-encoded oncogenic PIAS1 variant blocks immunogenic ferroptosis by modulating the balance between SUMOylation and phosphorylation of STAT1. Mol. Cancer 23 (1), 207. 10.1186/s12943-024-02124-6 39334380 PMC11438063

[B130] Zhang C. Lin M. Wu R. Wang X. Yang B. Levine A. J. (2011). Parkin, a p53 target gene, mediates the role of p53 in glucose metabolism and the Warburg effect. Proc. Natl. Acad. Sci. U. S. A. 108 (39), 16259–16264. 10.1073/pnas.1113884108 21930938 PMC3182683

[B131] Zhang Y. Qian Y. Zhang J. Yan W. Jung Y. S. Chen M. (2017). Ferredoxin reductase is critical for p53-dependent tumor suppression *via* iron regulatory protein 2. Genes Dev. 31 (12), 1243–1256. 10.1101/gad.299388.117 28747430 PMC5558926

[B132] Zhang C. Shang G. Gui X. Zhang X. Bai X. C. Chen Z. J. (2019). Structural basis of STING binding with and phosphorylation by TBK1. Nature 567 (7748), 394–398. 10.1038/s41586-019-1000-2 30842653 PMC6862768

[B133] Zhang S. Kang L. Dai X. Chen J. Chen Z. Wang M. (2022a). Manganese induces tumor cell ferroptosis through type-I IFN dependent inhibition of mitochondrial dihydroorotate dehydrogenase. Free Radic. Biol. Med. 193 (1), 202–212. 10.1016/j.freeradbiomed.2022.10.004 36228830

[B134] Zhang W. Gong M. Zhang W. Mo J. Zhang S. Zhu Z. (2022b). Thiostrepton induces ferroptosis in pancreatic cancer cells through STAT3/GPX4 signalling. Cell Death Dis. 13 (7), 630. 10.1038/s41419-022-05082-3 35859150 PMC9300693

[B135] Zhang W. Wang J. Liu Z. Zhang L. Jing J. Han L. (2022c). Iron-dependent ferroptosis participated in benzene-induced anemia of inflammation through IRP1-DHODH-ALOX12 axis. Free Radic. Biol. Med. 193 (1), 122–133. 10.1016/j.freeradbiomed.2022.10.273 36244588

[B136] Zhang Z. Tang J. Song J. Xie M. Liu Y. Dong Z. (2022d). Elabela alleviates ferroptosis, myocardial remodeling, fibrosis and heart dysfunction in hypertensive mice by modulating the IL-6/STAT3/GPX4 signaling. Free Radic. Biol. Med. 181, 130–142. 10.1016/j.freeradbiomed.2022.01.020 35122997

[B137] Zhang C. Hao H. Wang Y. Mu N. Jiang W. Zhang Z. (2023a). Intercellular mitochondrial component transfer triggers ischemic cardiac fibrosis. Sci. Bull. (Beijing) 68 (16), 1784–1799. 10.1016/j.scib.2023.07.030 37517989

[B138] Zhang W. Xu M. Chen F. Su Y. Yu M. Xing L. (2023b). Targeting the JAK2-STAT3 pathway to inhibit cGAS-STING activation improves neuronal senescence after ischemic stroke. Exp. Neurol. 368, 114474. 10.1016/j.expneurol.2023.114474 37419174

[B139] Zhang J. Zhu Q. Peng Z. Li X. J. Ding P. F. Gao S. (2024). Menaquinone-4 attenuates ferroptosis by upregulating DHODH through activation of SIRT1 after subarachnoid hemorrhage. Free Radic. Biol. Med. 210, 416–429. 10.1016/j.freeradbiomed.2023.11.031 38042225

[B140] Zhang L. Wang J. Deng W. Gui F. Peng F. Zhu Q. (2025). Solamargine induces hepatocellular carcinoma cell apoptosis and ferroptosis *via* regulating STAT1/MTCH1 axis. Biochem. Genet. 63 (1), 210–224. 10.1007/s10528-024-10749-x 38429602

[B141] Zhao Z. Ma Z. Wang B. Guan Y. Su X. D. Jiang Z. (2020). Mn^2+^ directly activates cGAS and structural analysis suggests Mn^2+^ induces a noncanonical catalytic synthesis of 2'3'-cGAMP. Cell Rep. 32 (7), 108053. 10.1016/j.celrep.2020.108053 32814054

[B142] Zhao J. Yi Z. Deng G. Li Y. Li J. Qin M. (2024a). STING modulates iron metabolism to promote liver injury and inflammation in acute immune hepatitis. Free Radic. Biol. Med. 210, 367–377. 10.1016/j.freeradbiomed.2023.11.038 38052276

[B143] Zhao Q. X. Yan S. B. Wang F. Li X. X. Shang G. K. Zheng Z. J. (2024b). STING deficiency alleviates ferroptosis through FPN1 stabilization in diabetic kidney disease. Biochem. Pharmacol. 222, 116102. 10.1016/j.bcp.2024.116102 38428828

[B144] Zhicheng J. Yongqian L. Peixuan W. Kai Y. Mengyu S. Wen C. (2024). ErZhiTianGui Decoction alleviates age-related ovarian aging by regulating mitochondrial homeostasis and inhibiting ferroptosis. J. Ovarian Res. 17 (1), 12. 10.1186/s13048-023-01341-9 38200521 PMC10777630

[B145] Zhong B. Zhang L. Lei C. Li Y. Mao A. P. Yang Y. (2009). The ubiquitin ligase RNF5 regulates antiviral responses by mediating degradation of the adaptor protein MITA. Immunity 30 (3), 397–407. 10.1016/j.immuni.2009.01.008 19285439

[B146] Zhong Y. Zhang W. Yu H. Lin L. Gao X. He J. (2022). Multi-platform-based characterization of ferroptosis in human colorectal cancer. iScience 25 (8), 104750. 10.1016/j.isci.2022.104750 35942097 PMC9356096

[B147] Zhou T. J. Zhang M. M. Liu D. M. Huang L. L. Yu H. Q. Wang Y. (2024). Glutathione depletion and dihydroorotate dehydrogenase inhibition actuated ferroptosis-augment to surmount triple-negative breast cancer. Biomaterials 305, 122447. 10.1016/j.biomaterials.2023.122447 38154441

[B148] Zhu M. Kim J. Deng Q. Ricciuti B. Alessi J. V. Eglenen-Polat B. (2023a). Loss of p53 and mutational heterogeneity drives immune resistance in an autochthonous mouse lung cancer model with high tumor mutational burden. Cancer Cell 41 (10), 1731–1748.e8. 10.1016/j.ccell.2023.09.006 37774698 PMC10693909

[B149] Zhu M. Peng L. Huo S. Peng D. Gou J. Shi W. (2023b). STAT3 signaling promotes cardiac injury by upregulating NCOA4-mediated ferritinophagy and ferroptosis in high-fat-diet fed mice. Free Radic. Biol. Med. 201, 111–125. 10.1016/j.freeradbiomed.2023.03.003 36940731

[B150] Zhu Q. Dai Q. Zhao L. Zheng C. Li Q. Yuan Z. (2024a). Novel dual inhibitors of PARP and HDAC induce intratumoral STING-mediated antitumor immunity in triple-negative breast cancer. Cell Death Dis. 15 (1), 10. 10.1038/s41419-023-06303-z 38182579 PMC10770036

[B151] Zhu Y. Jiang C. He J. He C. Zhou X. Huang X. (2024b). Cirbp suppression compromises DHODH-mediated ferroptosis defense and attenuates hypothermic cardioprotection in an aged donor transplantation model. J. Clin. Invest. 134 (9), e175645. 10.1172/jci175645 38690728 PMC11060748

